# Cardiorespiratory dynamics during respiratory maneuver in athletes

**DOI:** 10.3389/fnetp.2023.1276899

**Published:** 2023-10-30

**Authors:** Oleksandr Romanchuk

**Affiliations:** Department of Medical Rehabilitation, Ukrainian Research Institute of Medical Rehabilitation and Resort Therapy of the Ministry of Health of Ukraine, Odesa, Ukraine

**Keywords:** breathing maneuver, heart rate variability, systolic blood pressure variability, diastolic blood pressure variability, volume respiration variability, hemodynamics, athletes

## Abstract

**Introduction:** The modern practice of sports medicine and medical rehabilitation requires the search for subtle criteria for the development of conditions and recovery of the body after diseases, which would have a prognostic value for the prevention of negative effects of training and rehabilitation tools, and also testify to the development and course of mechanisms for counteracting pathogenetic processes in the body. The purpose of this study was to determine the informative directions of the cardiorespiratory system parameters dynamics during the performing a maneuver with a change in breathing rate, which may indicate the body functional state violation.

**Methods:** The results of the study of 183 healthy men aged 21.2 ± 2.3 years who regularly engaged in various sports were analyzed. The procedure for studying the cardiorespiratory system included conducting combined measurements of indicators of activity of the respiratory and cardiovascular systems in a sitting position using a spiroarteriocardiograph device. The duration of the study was 6 min and involved the sequential registration of three measurements with a change in breathing rate (spontaneous breathing, breathing at 0.1 Hz and 0.25 Hz).

**Results:** Performing a breathing maneuver at breathing 0.1 Hz and breathing 0.25 Hz in comparison with spontaneous breathing leads to multidirectional significant changes in heart rate variability indicators–TP (ms^2^), LF (ms^2^), LFHF (ms^2^/ms^2^); of blood pressure variability indicators–TP_DBP_ (mmHg^2^), LF_SBP_ (mmHg^2^), LF_DBP_ (mmHg^2^), HF_SBP_ (mmHg^2^); of volume respiration variability indicators - LF_R_, (L×min^-1^)^2^; HF_R_, (L×min^-1^)^2^; LFHF_R_, (L×min^-1^)^2^/(L×min^-1^)^2^; of arterial baroreflex sensitivity indicators - BR_LF_ (ms×mmHg^-1^), BR_HF_ (ms×mmHg^-1^). Differences in indicators of systemic hemodynamics and indicators of cardiovascular and respiratory systems synchronization were also informative.

**Conclusion:** According to the results of the study, it is shown that during performing a breathing maneuver with a change in the rate of breathing, there are significant changes in cardiorespiratory parameters, the analysis of which the increments made it possible to determine of the changes directions dynamics, their absolute values and informative limits regarding the possible occurrence of the cardiorespiratory interactions dysregulation.

## 1 Introduction

The modern practice of sports medicine and medical rehabilitation requires the search for subtle criteria for the development of conditions and recovery of the body after diseases, which would have a prognostic value for the prevention of negative effects of training and rehabilitation tools, as well as testify to the development and course of mechanisms for counteracting pathogenetic processes in the body (Kellmann et al., 2018). The complexity of this task in many cases is due to the feasibility of detecting minimal changes, which are often not clinically significant (Alchinova and Karganov, 2021; Guzii and Romanchuk, 2021b; Romanchuk et al., 2023). Given the adaptive processes that occur in the body, this occurs at the level of certain functional stresses in various systems and organs of the body (Nicolò et al., 2020). Of course, the modern focus of medicine on the evidence of certain signs of conditions and diseases significantly increases the requirements for the search and further consideration of a number of functional parameters of the body’s activity (Bernaciková et al., 2019). Standardization of modern diagnostic and therapeutic methods significantly increases the efficiency of diagnosis, treatment, and rehabilitation (Pinna et al., 2017), but has certain problems regarding a specific person.

This is due to the fact that the existing arsenal of modern functional diagnostics in the vast majority of cases is based on the identification of markers of pathology, that is, it is reduced to the detection of signs that characterize the formation of a pathological trace in the body (Guenette and Sheel, 2007; Illigens and Gibbons, 2019). At the same time, the available experience of prognostic medicine proves that even with reliable identification of pathological markers, the final result of an individual prognosis of the course of an organism’s pathology and its recovery is quite problematic (Noskin et al., 2020).

These difficulties can be overcome by implementing into the practice of functional diagnostics methods of combined instrumental research of various functions, which allow establishing individual variants of intersystem interactions at rest and in the dynamics of certain influences (Malliani et al., 1994; Dick et al., 2014; Harford et al., 2019; Abreu et al., 2023).

The level of individual functions correlation at the intra- and inter-system levels is of great importance in multi-functional studies. In this regard, only objective data can be a condition for reliable prediction of certain dysregulations, which means risks of susceptibility to the development of diseases, pathological processes during sports, or restoration of functionality after injuries and diseases (de la Sierra et al., 2020; Noskin et al., 2020; Angelova et al., 2021; Da Silva et al., 2023).

Cardio-respiratory interaction, which occurs at many levels of the nervous system and includes the coordinated regulation of respiratory, cardiovagal, and sympathetic influences, occupies a leading place in understanding the mechanisms of dysregulation (Cottin et al., 1999; Malpas, 2010; Javorka et al., 2020). Many neurons in each of the two ventrolateral medullary networks (respiratory and autonomic) are differentiated and directly regulated by oligosynaptic input signals from chemoreceptors, baroreceptors, pulmonary mechanoreceptors, and their control is carried out through the formation of patterns of cardiovascular and respiratory interactions in the cerebral cortex, hypothalamus, pons (Garcia et al., 2013; Porta et al., 2016). Therefore, the determining value for their activity estimation, even at the level of the function implementation, is given to the simultaneous registration of indicators of their activity, which significantly reduces the error when establishing their interaction (Alchinova and Karganov, 2021; Romanchuk et al., 2023).

Maximizing an athlete’s performance is not only part of the training process. It also depends on an optimal balance between training and recovery, which is the key to preventing maladaptation, which can occur due to the accumulation of psychological and physiological stresses caused by the training and training load (Kellmann et al., 2018). From these positions, it is necessary to mention that physical exertion, which is an undeniable condition for increasing the level of training, on the other hand, can cause physical overload and be accompanied by adequate and inadequate recovery (Dupuy et al., 2018). In the first case, this is a condition for training growth, and in the second case, it is a prerequisite for the formation of pre-pathological states of functional (Bellinger, 2020; Nicolò et al., 2020) and non-functional overreaching (Nederhof et al., 2008; Meeusen et al., 2013), and as well as the development of overtraining (Grivas, 2018; Armstrong et al., 2022). At the same level as the neuromuscular apparatus, the leading role in the formation of these conditions belongs to the autonomic and cardiorespiratory systems (Baumert et al., 2006).

It is well known that deep breathing with a frequency of 0.1 Hz is characterized by an increase in respiratory volume and can increase the excursion of the diaphragm (Vostatek et al., 2013; Stromberg et al., 2015; Russo et al., 2017; Fuller et al., 2022), promotes more effective ventilation and oxygenation of blood due to the involvement of more alveoli, and also reduces alveolar dead space (Bilo et al., 2012). A decrease in chemoreflex sensitivity has been shown (Bernardi et al., 2001; Sampaio et al., 2012). Involvement of this extracardiac circulatory factor contributes to an increase in venous return, filling of the right heart chambers and, accordingly, an increase in stroke volume and cardiac output (Hsieh et al., 2003; Byeon et al., 2012; Elstad, 2012; Dick et al., 2014). The effect of synchronizing pulse oscillations of systolic and diastolic blood pressure with heart rhythm is noted (Hsieh et al., 2003; Romanchuk, 2005; Romanchuk, 2013; Ovadia-Blechman et al., 2017). An improvement in capillary flow is likely (Ovadia-Blechman et al., 2017). The effects of increasing HRV and BPV are quite obvious (Bernardi et al., 2001; Radaelii et al., 2004; Romanchuk, 2005; Sin et al., 2010; Chang et al., 2015). Mean arterial pressure may decrease (Radaelii et al., 2004; Dick et al., 2014). The LF-component of HRV and baroreflex sensitivity increase (Eckberg, 2003; Beda et al., 2014; Guzii and Romanchuk, 2016; Romanchuk and Guzii, 2017; Guzii et al., 2019). Resonantly, 0.1 Hz breathing increases RSA activity (Bernardi et al., 2001; Vaschillo et al., 2006). The efficiency of pulmonary gas exchange improves (Hayano et al., 1996; Mortola et al., 2016), the economy of heart work is noted (Ben-Tal et al., 2014), as well as the buffering of blood pressure fluctuations (Hsieh et al., 2003; Elstad M., 2012). On the part of the autonomic nervous system, the effects of increased activity and tone of the parasympathetic link are observed (Chang et al., 2015). The optimization of acetylcholine release and hydrolysis in the SA node (Wang et al., 2013) and the strengthening of phase modulation of sympathetic activity (Limberg et al., 2013) were noted, contributing to the improvement of orthostatic reactions (Vidigal et al., 2016). That is, deep breathing with a frequency of 0.1 Hz allows you to determine the structure of the body’s systems under the imposed parasympathetic influence.

Deep breathing 15 times per minute (0.25 Hz) in an imposed rhythm for 2 min can cause the initial signs of hyperventilation and affect various systems and tissues of the body, modifying the manifestations of sympathoadrenal activation (Hornsveld et al., 1995; Krohn et al., 2023). The latter was shown when testing with hyperventilation for 100 s at a rhythm of 0.33 Hz (Kox et al., 2014). However, hyperventilation at a rate of 0.25 Hz for 2 min is quite gentle, although it can also cause the development of certain symptoms. Among the signs that we obtained in previous studies: an increase in HR (min^-1^), resonant predominance of the HF component of HF (ms^2^), HF_SBP_ (mmHg^2^) and HF_DBP_ (mmHg^2^) (Romanchuk, 2013; Guzii et al., 2019), a decrease in BR_LF_ (ms×mmHg^-1^), BR_HF_ (ms×mmHg^-1^) at rest and during the recovery period after exercise (Guzii and Romanchuk, 2016), changes in systemic hemodynamics (Romanchuk, 2013) If we talk about hyperventilation tests in general, their classical performance with a duration of up to 3 min can stimulate the appearance of symptoms (King, 1988), which can cause a deterioration in wellbeing, or even contribute to the development of conditions that will require emergency care (Gardner, 1996; Gilbert, 1999). In view of the above, we proposed a procedure for performing a breathing maneuver, which is gentle on reactions, but it allows you to detect the body’s reactivity to influences that stimulate, at least, the activation of the sympathetic and parasympathetic links of the autonomic nervous system.

In our opinion, the study of cardiorespiratory relationships is of significant importance in determining the functional state of the human body and will contribute to the development of new approaches to the diagnosis of changes in the body of patients, practically healthy persons and athletes.

The main hypothesis of this study was that the changes that occur in the cardiorespiratory system during the performance of a breathing maneuver with a change in breathing rate can have a diagnostic value in determining the current functional state of an athlete’s body.

The purpose of this study was to determine the informative directions of the dynamics of the cardiorespiratory system parameters during a maneuver with a change in breathing rate, which may indicate a violation of the functional state of the body.

## 2 Materials and methods

### 2.1 Study subjects

This study was conducted in the limits scientific programs of departments of Exercises Medicine and Sports Medicine of South Ukrainian National Pedagogical University (September 2012 - July 2016) and Sports Medicine of Lviv State University of Physical Culture (January 2021), on different sports bases of Odesa, Lviv and of team Ukraine. We analyzed the results of the study of 183 healthy men aged 21.2 ± 2.3 years who regularly engaged in various sports (Table 1), did not complain of any problems in the state of the body, did not have acute diseases and were allowed to participate in sports according to the results of the last medical examination. The length of time in sports ranged from 3 to 15 years, the level of sportsmanship ranged from a candidate for master of sports to champions of Ukraine, Europe, the World, and the Olympic Games. All examinations were carried out in the morning, 2–3 h after a light breakfast. On the eve of the study, all participants were instructed by trainers to avoid consumption of stimulant beverages (coffee, green tea, energy drinks) before the examination. Taking into account that the examination was carried out in different periods of the annual training cycle, the main condition for admission to the study was the absence of intense and prolonged physical load the day before.

**TABLE 1:** Distribution of athletes by sports and place of investigate.

Kind of sport	N	Examination place	
Long distance runners	2	“Olympyets”, Odesa School of Higth Sport Mastery	School doctor’s office
Middle distance runners	6	“Olympyets”, Odesa School of Higth Sport Mastery	School doctor’s office
Rowers on kayaks	5	FSC “Khimik”, Pivdennyy water and rowing base	Base doctor’s office
Rowers on canoes	6	FSC “Khimik”, Pivdennyy water and rowing base	Base doctor’s office
Table tennis players	14	TTC “Burevisnyk”, Odesa	TTC doctor’s office
Boxing	33	Olympic educational sport center “Koncha Zaspa”	A small gym
Karate	12	SC “SUNPU”, Odesa	SC doctor’s office
Freestyle wrestling	11	SC “SUNPU”, Odesa	SC doctor’s office
Judo	3	SC “SUNPU”, Odesa	SC doctor’s office
Greco-Roman wrestling	5	SC “SUNPU”, Odesa	SC doctor’s office
Volleyball	13	SC “SUNPU”, Odesa	SC doctor’s office
Water polo	14	WPC “Dinamo”, Lviv	WPC doctor’s office
Handball	9	SC “SUNPU”, Odesa	SC doctor’s office
Soccer	35	FC “Chernomorets”, Odesa	Base doctor’s office
Gymnasts	3	SC “SUNPU”, Odesa	SC doctor’s office
Acrobats	2	SC “SUNPU”, Odesa	SC doctor’s office
Shooters	10	“Olympyets”, Odesa School of Higth Sport Mastery	School doctor’s office

Abbreviations: SC, sports club; FC, Soccer club; WPC, Water polo club; TTC, table tennis club; SUNPU, South Ukrainian National Pedagogical University.

**FIGURE 1:**
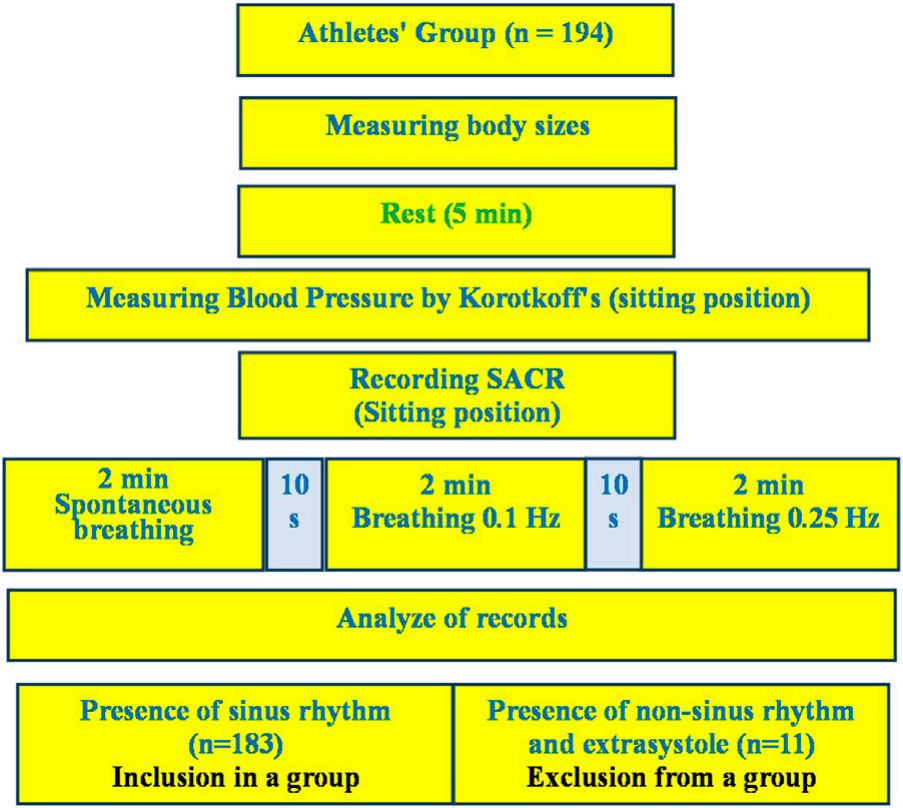
Design of research.

The only condition for exclusion from the analysis of the examination results was the presence of heart rhythm disturbances in the form of extrasystoles and non-sinus rhythm, which was establishing during the preliminary analysis of the examination records. A total of 11 such cases were registered. From 11 mentioned cases there were extrasystoles in three cases and non-sinus rhythm in two cases in rest. In six cases the extrasystoles appeared in breathing 6 times per minute. They are not included in the analysis group (Figure 1). The athletes had no any complaints and discomfort during and after the breathing maneuver.

This study was approved by the Ethics Committee of the South Ukrainian National Pedagogical University (No. 121), by the Ethics Committee of the Lviv State University of Physical Culture (No. 33-16). All athletes were informed about the study and signed an informed consent form before the trial.

### 2.2 Procedure of study

**FIGURE 2:**
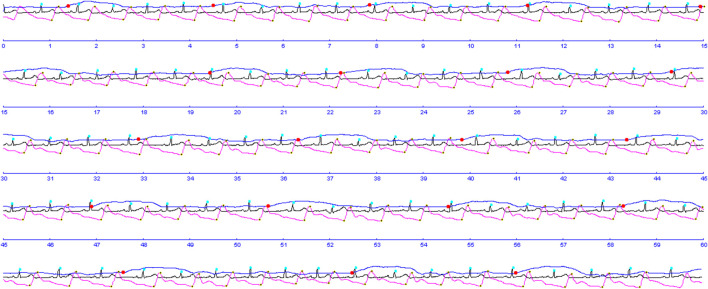
Example of record with SACR (fragment).

The procedure for studying the cardiorespiratory system included conducting combined measurements of indicators of activity of the respiratory and cardiovascular systems in a sitting position using a spiroarteriocardiograph (SACR) device. The duration of the study was 6 min and involved the sequential registration of three measurements (2 min each) with a change in breathing rate. During the first 2 min, SACR indicators were recorded during normal spontaneous breathing (SR) (Figure 2), during the second 2 min–during controlled breathing 6 times per minute (5 s inhalation, 5 s exhalation) (CR_6_), during the third 2 min–during controlled breathing 15 once per minute (2 s inhalation, 2 s exhalation) (CR_15_).

### 2.3 Method

To determine cardiorespiratory station the “Spiroarteriocardiorhythmograph” device was applied (SACR; Intoks Company, St. Petersburg). The device combines three certain methods of physiological studies into an integrated hardware complex, which makes it possible to achieve a fundamentally new quality of measurements, that is, simultaneous recording of HRV and ВPV at different stages of the respiratory act (Pivovarov, 2006; Pivovarov, 2011).

ECG recording in 1 lead allowed to determine the indicators of heart rate variability (HRV) according to the spectral analysis of the sequence of RR intervals is total power (ТР, ms^2^), power in the very low frequency range (VLF, ms^2^), power in the low frequency range (LF, ms^2^) and power in the high frequency range (HF, ms^2^) and their derivatives (LFn, n. u., HFn, n. u., LF/HF); according to the math analysis of the sequence of RR intervals is ABI (autonomic balance index, c. u.), SRAI (subcortical regulation adequacy indicator, c. u.), ARI (autonomic regulation index, c. u.), SI (stress index, c. u.), SDANN (standard deviation of the values of cardiointervals, ms), RMSSD (square root of the sum of squares of the differences in the values of consecutive pairs of normal intervals, ms), pNN50 (the percentage of NN50 from the total number of consecutive pairs of intervals that differ by more than 50 milliseconds, obtained over the entire time recording, %) (Malik et al., 1996); according to cardiointervalometry–to define the heart rate (HR, min^-1^), averages durations and intervals of PQRST-complex–P (s), PQ (s), QRS (s), QT (s), QTC (s), ST (n.u.); indicators of systemic hemodynamics (Kim et al., 2005)—end-diastolic volume (EDV, cm^3^), end-systolic volume (ESV, cm^3^), stroke volume (SV, cm^3^), cardiac output (CO, dm^3^), stroke index (SI, cm^3^×m^-2^), cardiac index (CI, dm^3^×m^-2^), general peripheral vascular resistance (GPVR, dyn/s/cm^−5^); according to the pulse wave recording with the help of a photoplethysmographic sensor on the finger by the Penaz method (Penáz, 1973; Wesseling, 1990), blood pressure (SBP, mmHg; DBP, mmHg) and its variability (SBPV and DBPV) in ranges similarly to HRV were determined a total power of SBPV and DBPV (ТР_SBP_, mmHg^2^ and ТР_DBP_, mmHg^2^), power in the very low-frequency range (VLF_SBP_, mmHg^2^ and VLF_DBP_, mmHg^2^), power in the low-frequency range (LF_SBP_, mmHg^2^ and LF_DBP_, mmHg^2^) and power in the high-frequency range (HF_SBP_, mmHg^2^ and HF_DBP_, mmHg^2^) and their derivatives–LF_SBP_n, n. u., HF_SBP_n, n. u., LF/HF_SBP_, LF_DBP_n, n. u., HF_DBP_n, n. u., LF/HF_DBP_ (Parati et al., 1995; Pinna et al., 1996; Karemaker, 1997; Rosei et al., 2020). Additionally, by using the spectral method we determined the index of arterial baroreflex sensitivity (BR, ms/mmHg)—α-coefficient, that was calculated in high (BR_HF_) and low (BR_LF_) frequencies ranges (Karemaker and Wesseling, 2008; Rydlewska et al., 2010; Papaioannou et al., 2019).

$${\mathrm{BR}}_{\mathrm{LF}}=\sqrt{{\mathrm{LF}}_{\mathrm{HRV}}/{\mathrm{LF}}_{\mathrm{SBP}}}$$

$${\mathrm{BR}}_{\mathrm{HF}}=\sqrt{{\mathrm{HF}}_{\mathrm{HRV}}/{\mathrm{HF}}_{\mathrm{SBP}}}$$

The ultrasonic sensor of the SACR device allows to measure flows of air on inspiration and expiration and to define the average parameters of a respiration pattern (RP): duration of inspiratory (T_I_, s), duration of expiratory (T_E_, s), tidal volume (V_T_, L), volumetric inspiratory velocity (V_I_, L×s^-1^), volumetric expiratory velocity (V_E_, L×s^-1^), the fraction of inspiration in the respiratory cycle (T_I_/(T_I_ + T_E_) (c.u.), as well as the volume of minute respiration (V); and calculate the parameters of volume respiration variability (VRV): total power of respiration (TP_R_, (L×min^-1^)^2^), respiration power in the very low-frequency range (VLF_R_ (L×min^-1^)^2^), respiration power in the low-frequency range (LF_R_ (L×min^-1^)^2^) and respiration power in the high frequency range (HF_R_ (L×min^-1^)^2^) and their derivatives–LF_R_n, HF_R_n, LF/HF_R_–in n. u. (Romanchuk and Guzii, 2017; Bazhora and Romanchuk, 2018; Romanchuk and Guzii, 2020).

**FIGURE 3:**
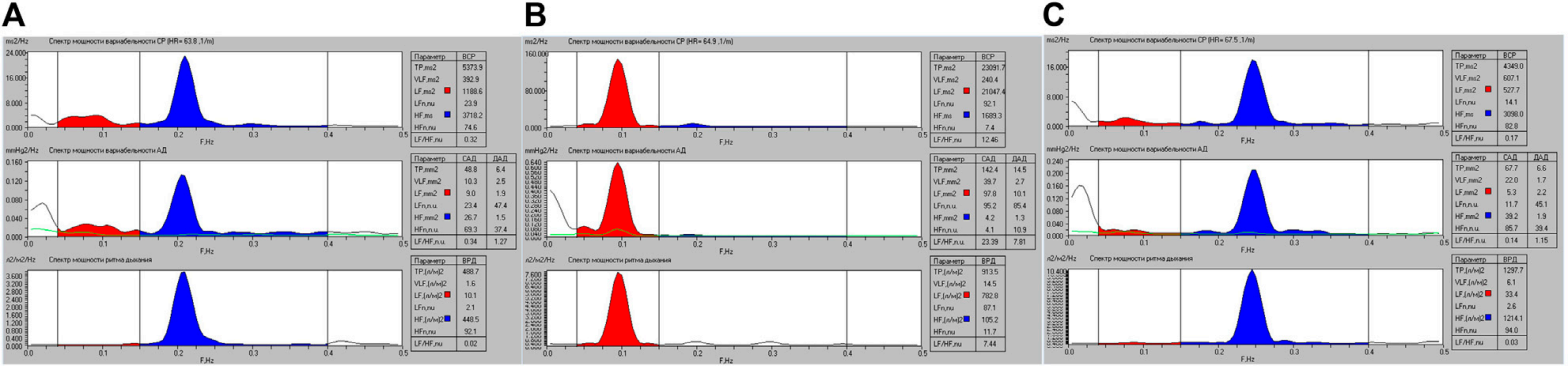
Graphic representation of the spectral powers of HR, SBP, DBP and breathing variability for athlete K. when performing a breathing maneuver with SR **(A)**, with CR_6_ **(B)** and with CR_15_ **(C)**.

**FIGURE 4:**
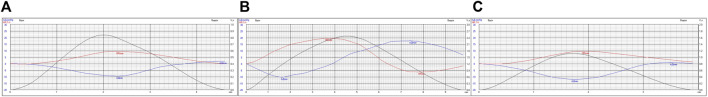
Variability of absolute values of HR (min^-1^) and SBP (mmHg) in the respiratory cycle of athlete K. when performing a breathing maneuver with SR **(A)**, CR_6_ **(B)**, CR_15_ **(C)**.

As an example, we will show differences in Fourier transformation spectra of the functions studied which were measured for the same person performing a breathing maneuver with spontaneous respiration (Figures 3A, 4A), controlled respiration (СR) at the rate of 6 times per minute (Figures 3B, 4B) and 15 times per minute (Figures 3C, 4C), further on CR_6_ and CR_15_, respectively. In more previous studies we have shown that within the range from 6 to 10 breaths per minute there is an inversely proportional relationship between SR and HRV and BPV values (Romanchuk, 2005; Romanchuk et al., 2011), and at SR_15_ HRV and BPV are stabilized.

These data are confirmed by the results shown in Figure 4, where the average versions of HR and SBP variability are shown within the limits of the respiratory cycle.

Indicators of frequency and volume synchronization of the cardio-respiratory system were also calculated–Hildebrandt index (HI) and VSI (Hildebrandt, 1953; Noskin et al., 2018).

$$\left.HI=HR\;\left(\min^{-1}\right)/{RR(\min}^{-1}\right)$$

$$VSI=CO\;\left({\mathrm{dm}}^{3}\times \min^{-1}\right)/V\;\left(L\times \min^{-1}\right)$$

### 2.4 Statistical analysis

The processing of the received results was carried out with the help of STATISTICА program for Windows (version 10.0), Microsoft Excel 2012. The data obtained are presented as a median with 25%–75% (Q_1_; Q_3_) percentiles. Differences between initial and subsequent measurements were taken via Wilcoxon matched-pairs test.

## 3 Results

### 3.1 Morphofunctional data result

Table 2 presents the morphofunctional parameters of the examined group of athletes, the results of which were subjected to further analysis. As can be seen from the results of the examination, in general, the group has a sufficiently high level of physical development in most parameters.

**TABLE 2:** Morphofunctional parameters of the examined group of athletes, Med (Q1; Q3), n = 183.

Indicator	Values
Body weigh, kg	73.0 (68.0; 80.0)
Body length, cm	178.0 (173.0; 182.0)
BMI, kg×m^-2^	23.5 (22.0; 24.8)
Body square, m^2^	1.90 (1.82; 2.01)
Body length (sitting), cm	93.0 (91.0; 95.0)
Fat, %	14.5 (11.3; 17.3)
Chest circumference (pause), cm	97.0 (93.0; 102.0)
Chest excursion, cm	8.0 (7.0; 9.0)
VC, ml	4,900.0 (4,500.0; 5,500.0)
δVC, %	9.56 (−1.03; 21.04)
VI, ml×kg^-1^	66.7 (60.6; 72.9)
Test Shtange, s	76.0 (63.0; 93.0)
Test Genchee, s	40.0 (32.0; 50.0)
SBP, mmHg	114 (110.0; 120.0)
DBP, mmHg	70.0 (60.0; 80.0)
PBP, mmHg	50.0 (40.0; 50.0)

Abbreviations: BMI, body mass index; VC, vital lung capacity; δVC, different between vital lung capacity and proper vital lung capacity in percentage; VI, vital index; SBP, systolic blood pressure (by Korotkoff); DBP, diastolic blood presure (by Korotkoff); PBP, pulse blood pressure (by Korotkoff).

### 3.2 Breathing maneuver and indicators of breathing pattern and volume variability

For a clearer further characterization of the combined changes that occur in the cardiorespiratory system, it is first appropriate to describe the changes that occur in the breathing pattern during the breathing maneuver. It should be noted that the SACR device includes an ultrasonic air movement sensor, so the results obtained during the maneuver are accurate in relation to the flows of inhaled and exhaled air. In Table 3 presents the results of measuring the main indicators of pattern breathing, which prove their significant differences when performing a breathing maneuver. The differences apply to all parameters, which are quite expected. Tidal volume (V_T_, L) is greatest at CR_6_, which characterizes the depth of breathing that reaches one-third of VC (L), increasing threefold compared to SR. At the same time, V (L×min^-1^) increases by an average of 1.5 times. On the other hand, at CR_15_, there is a 1.5-fold increase in V_T_ (L) on the background of an almost two-fold increase in V (L×min^-1^). A significant increase in VO_2_ (L×min^-1^), which is maximal at CR_15_, is also indicative. That is, the data of patterned breathing prove that the main irritating effect in CR_6_ is deep fluid breathing, which can suppress the efferent postganglionic activity of the sympathetic nerve, and in CR_15_ it is moderately deep, more often rhythmic breathing, which is accompanied by a more significant V (L×min^-1^) and VO_2_ (L×min^-1^), which can stimulate signs of hyperventilation. At the same time, this effect can be facilitated by a more significant increase in air flow rates during inhalation (V_T_/T_I_, L×s^-1^) and exhalation (V_T_/T_E_, L×s^-1^) at CR_15_ (Table 3). Probably, such testing can be useful in the practice of diagnosing functional and non-functional overreaching syndromes that can occur in highly qualified athletes (Nicolò et al., 2020).

**TABLE 3:** Indicators of breathing pattern of the examined group of athletes during the breathing maneuver, Med (Q1; Q3), n = 183.

Indicator	SR	CR_6_	CR_15_	p		
				SR-CR_6_	SR-CR_15_	CR_6_-CR_15_
Ti, s	1.76 (1.44; 2.09)	4.98 (4.82; 5.12)	1.92 (1.78; 2.06)	0.000	0.004	0.000
Te, s	2.43 (2.04; 3.06)	5.02 (4.90; 5.14)	2.07 (1.95; 2.20)	0.000	0.000	0.000
V_T_, L	0.570 (0.440; 0.700)	1.700 (1.220; 2.270)	0.890 (0.660; 1.280)	0.000	0.000	0.000
V_T_ /T_E_, L×s^-1^	0.22 (0.17; 0.28)	0.31 (0.24; 0.43)	0.40 (0.29; 0.60)	0.000	0.000	0.000
V_T_/T_I_, L×s^-1^	0.32 (0.27; 0.38)	0.41 (0.29; 0.55)	0.53 (0.38; 0.71)	0.000	0.000	0.000
T_I/_ /T_TOT_	0.41 (0.39; 0.44)	0.49 (0.47; 0.51)	0.48 (0.46; 0.51)	0.000	0.000	0.541
RR, min^-1^	14.2 (12.0; 16.8)	6.2 (6.0; 6.4)	15.2 (15.0; 15.3)	0.000	0.000	0.000
V, L×min^-1^	7.818 (6.307; 9.583)	11.050 (7.930; 14.755)	13.740 (10.109; 19.447)	0.000	0.000	0.000
VO_2_,L×min^-1^	0.360 (0.290; 0.441)	0.508 (0.365; 0.679)	0.632 (0.465; 0.895)	0.000	0.000	0.000

Abbreviations: Ti, mean inspiratory time; Te, mean expiratory time; V_T_, tidal volume; V_T_/T_E_, mean exspiratory flow; V_T_/T_I_, mean inspiratory flow; T_I_//T_TOT_, mean inspiratory duty cycle; RR, respiratory rate; V, minute ventilation; VO_2_, oxygen uptake.

Breathing regulation has significant individual and situational variability. It is the latter that stops attempts to investigate spontaneous breathing, and also translates scientific research into researching the perfusion-ventilation capabilities of the respiratory apparatus. Of course, this is important from the point of view of understanding oxygen supply and gas exchange, but it does not allow to fully characterizing the regulatory effects, being limited to chemo- and mechanoreceptor mechanisms (Byeon et al., 2012; Dempsey and Smith, 2019). In recent years, the results of a number of studies conducted in laboratory conditions that analyze the central modulatory effects on breathing have appeared (Girin et al., 2021; Shams et al., 2021; Balban et al., 2023). They reveal new breathing control mechanisms. Our research allows us to state that an important component of the optimal functional state of the body is the mechanisms that ensure the volume respiration variability (VRV). After all, VRV indicators, which are calculated using spectral analysis, make it possible to distinguish between different frequencies effects, which can significantly supplement information on regulatory effects on the respiratory system (Romanchuk et al., 2022; Balban et al., 2023). On the other hand, these effects are transmitted to the functions of the cardiovascular system, gas exchange, metabolism (Censi et al., 2002; Beda et al., 2014; Fonoberova et al., 2014; Paprika et al., 2014; Brændholt et al., 2023). Also, the possibilities of controlled breathing are well known, which is often used to stabilize the neuropsychological state, in the treatment of various pathologies, etc. (Ashhad et al., 2022; Krohn et al., 2023). The latter, based on the principle of feedback, activates certain areas of the CNS (Balban et al., 2023), contributing to the formation of new functional systems that trigger optimization or recovery mechanisms (Paprika et al., 2014).

A well-known complex indicator that is equivalent to the body’s needs in various states is V (L×min^-1^), which is calculated as the product of RR (min^-1^) and V_T_ (L). Taking this into account, we proposed to use indicators of respiratory volume variability (VRV), which are closely related to V (L×min^-1^) (Romanchuk et al., 2011) and are derived from volumetric inspiratory rates, to assess the state of the respiratory system and exhalation, but simultaneously take into account the rhythmic and volumetric characteristics of breathing. Previously, during the examination of 1930 young men, the limits of normative values for SR were determined, which were within the 25-75 percentile limits for TP_R_, (L×min^-1^)^2^–290.0–635.0; VLF_R_, (L×min^-1^)^2^–1.3–4.8; LF_R_, (L×min^-1^)^2^–7.9–33.6; HF_R_, (L×min^-1^)^2^–207.4–547.5; LFHF_R_, (L×min^-1^)^2^/(L×min^-1^)^2^–0.025–0.150 (Guzii et al., 2019). As can be seen from Table 4 with SR, all indicators are within the specified regulatory limits. When performing a breathing maneuver, the indicators change significantly, this is compared with the pattern breathing data, which showed an increase in the volumetric rate of inhalation and exhalation at CR_6_ and CR_15_. As well as among the spectral indicators of heart rate and blood pressure, the LF_R_ indicator, (L×min^-1^)^2^, which was resonant with respect to the respiratory frequency of 0.1 Hz (CR_6_), which according to its contribution to the total spectral power (LF_R_n) increased from 4.7 (2.5; 16.6) n. u. to 85.0 (78.0; 88.0) n. u., and at a breathing frequency of 0.25 Hz (CR_15_) decreased to 1.8 (1.3; 2.5) n. u., *p* = 0.000, which was significantly lower than at SR. At the same time, according to absolute values (LF_R_, (L×min^-1^)^2^) at a breathing frequency of 0.25 Hz (CR_15_), it did not differ from SR. Actually, it is the development of the state of hyperventilation that can contribute to such a discrepancy in the changes of this indicator.

**TABLE 4:** Indicators of volume respiration variability of the examined athletes during the breathing maneuver, Med (Q_1_; Q_3_), n = 183.

Indicator	SR	CR_6_	CR_15_	p		
				SR-CR_6_	SR-CR_15_	CR_6_-CR_15_
TP_R_, (L×min^-1^)^2^	349.7 (234.1; 547.6)	655.4 (334.9; 1,246.1)	1,108.9 (533.6; 2,284.8)	0.000	0.000	0.000
VLF_R_, (L×min^-1^)^2^	2.9 (1.7; 4.0)	10.2 (6.8; 18.5)	5.3 (2.6; 10.2)	0.000	0.000	0.000
LF_R_, (L×min^-1^)^2^	16.0 (9.6; 56.3)	542.9 (259.2; 1,024.0)	20.3 (10.9; 38.4)	0.000	0.649	0.000
LF_R_n,n.u	4.7 (2.5; 16.6)	85.0 (78.0; 88.0)	1.8 (1.3; 2.5)	0.000	0.000	0.000
HF_R_, (L×min^-1^)^2^	278.9 (169.0; 445.2)	81.0 (47.7; 146.4)	1,062.8 (515.3; 2,199.6)	0.000	0.000	0.000
HF_R_n,n.u	87.3 (74.2; 92.3)	13.2 (10.2; 19.0)	95.3 (92.9; 96.7)	0.000	0.000	0.000
LFHF_R_, (L×min^-1^)^2^/(L×min^-1^)^2^	0.053 (0.029; 0.203)	6.452 (4.040; 8.585)	0.020 (0.014; 0.029)	0.000	0.000	0.000
IC_R_, (L×min^-1^)^2^/(L×min^-1^)^2^	0.065 (0.035; 0.249)	6.572 (4.113; 8.879)	0.025 (0.018; 0.035)	0.000	0.000	0.000

Abbreviations: TP_R_, dispersion of V_T_/T_E_, in a given time interval (total power) ≈≤ 0.4 hz; VLF_R_, power spectrum of V_T_/T_E_, in the very-low frequency range ≤0.04 Hz; LF_R_, power spectrum of V_T_/T_E_, in the low frequency range 0.04–0.15 Hz; LF_R_n, LF_R_/(TP_R_–VLF_R_)×100; spectrum power of V_T_/T_E_, in the low-frequency range in normalized units; HF_R_, power spectrum of V_T_/T_E_, in the high frequency range 0.15–0.4 hz; HF_R_n, HF_R_/(TP_R_–VLF_R_)×100; spectrum power of V_T_/T_E_, in the high-frequency range in normalized units; LFHF_R_, LF_R_[(L×min^-1^)^2^]/HF_R_[(L×min^-1^)^2^]; IC_R_, (LF_R_[(L×min^-1^)^2^]+VLF_R_[(L×min^-1^)^2^])/HF_R_[(L×min^-1^)^2^]; centralization index of respiration.

### 3.3 Breathing maneuver and heart rate variability

According to the data of the SACR study, the average duration of cardio intervals during the registration period is calculated. Usually, their adequate assessment is possible if there are no extrasystolic contractions. Therefore, at the preliminary stage of recording analysis, all cases with existing extrasystoles were excluded from further analysis. In Table 5 shows the changes in the analysis indicators of the PQRST complex during the breathing maneuver, which indicate a significant increase in HR (min^-1^) from SR through CR_6_ to CR_15_ from 67.2 (62.1; 76.9) through 71.0 (64.8; 78.8) to 76.9 (69.2; 89.3), *p* = 0.000. Similarly, there is an increase in the electrical systole of the ventricles (QTc, s) from 0.408 (0.395; 0.422) through 0.412 (0.400; 0.424) to 0.421 (0.408; 0.434), *p* = 0.000. At the same time, the indicator of ST deviation (n.u.) from the isoline significantly increases at CR_6_, and then at CR_15_ it remains unchanged. The more significant increase in HR (min^-1^) at CR_15_ is most likely a response to hyperventilation, which is associated with a more significant increase in V (L×min^-1^) and VO_2_ (L×min^-1^). As a criterion for deterioration of the contractile function of the heart, an increase in QTc (s) can be considered, although the deviation of ST (n.u.) from the isoline, which indicates signs of myocardial ischemia, is not significant in comparison with SR and CR_6_. Among other cardiointervals, no significant changes were noted during the breathing maneuver.

**TABLE 5:** Informative changes in the indicators of the PQRST complex of the examined group of athletes during a breathing maneuver, Med (Q_1_; Q_3_), n = 183.

Indicator	SR	CR_6_	CR_15_	p		
				SR-CR_6_	SR-CR_15_	CR_6_-CR_15_
HR, min^-1^	67.2 (62.1; 76.9)	71.0 (64.8; 78.8)	76.9 (69.2; 89.3)	0.000	0.000	0.000
QTc, s	0.408 (0.395; 0.422)	0.412 (0.400; 0.424)	0.421 (0.408; 0.434)	0.000	0.000	0.000
ST,n.u	0.089 (0.052; 0.136)	0.102 (0.053; 0.148)	0.103 (0.044; 0.152)	0.005	0.289	0.557

Abbreviations: HR, heart rate; QTc, electrical systole corrected (by Bazett).

Existing approaches to the classification of HRV indicators most often involve spectral, statistical and geometric analysis. In Table 6 shows the changes in spectral indicators of HRV. Changes in spectral parameters during controlled breathing at 0.1 Hz and 0.25 Hz are largely determined by the resonant effects of breathing on the activity of the sinus node. This is quite indicative in Figure 3 (Guzi et al., 2019). At the same time, the absolute values of HRV indicators also change. Among all the indicators, quite stable values (VLF, ms^2^) deserve attention, which differ from the spontaneous (SR) only at CR_6_, *p* = 0.001, and at CR_15_ they do not differ either from the original, *p* = 0.083, or from CR_6_, *p* = 0.142, which may reflect the stable activity of central regulatory systems and psychoemotional influences. Also noteworthy is the absence of differences in the absolute values of HF (ms^2^) at SR and CR_6_, *p* = 0.405, which may indicate the absence of activation of high-frequency influences during postganglionic suppression of the sympathetic nerve. Analyzing the changes in other indicators of the spectral analysis of HRV, it can be stated that at CR_6_ there is an increase in the general regulatory effects (TP, ms^2^) on the heart rhythm, while at CR_15_ the activity significantly decreases (Table 6). In this case, a more significant decrease occurs in the low frequency range of 1,204.1 (645.2; 2,642.0) at SR to 552.3 (306.3; 912.0) at CR_15_, *p* = 0.000, and in the high -frequency range from 1,664.6 (912.0); 2,745.8) at CR_15_, *p* = 0.000. This suggests that changes in HF (ms^2^) are more resistant to changes in respiratory rate than LF (ms^2^).

**TABLE 6:** Indicators of HRV of the examined athletes during the breathing maneuver, Med (Q1; Q3), n = 183.

Indicator	SR	CR_6_	CR_15_	p		
				SR-CR_6_	SR-CR_15_	CR_6_-CR_15_
TP, ms^2^	4,096 (2,450; 6,939)	18,879 (13,712; 25,824)	2,884 (1747; 4,900)	0.000	0.000	0.000
VLF, ms^2^	501.8 (249.6; 882.1)	655.4 (408.0; 1,049.8)	547.6 (262.4; 998.6)	0.001	0.083	0.142
LF, ms^2^	1,204.1 (645.2; 2,642.0)	15,775.3 (11,470.4; 20,050.5)	552.3 (306.3; 912.0)	0.000	0.000	0.000
LFn,n.u	42.0 (24.8; 60.3)	86.7 (81.9; 91.6)	28.1 (17.7; 42.5)	0.000	0.000	0.000
HF, ms^2^	1,664.6 (912.0; 3,364.0)	2043.0 (1,069.3; 3,636.1)	1,497.7 (645.2; 2,745.8)	0.405	0.001	0.000
HFn,n.u	55.4 (35.8; 72.3)	12.1 (7.6; 16.7)	67.7 (53.3; 78.6)	0.000	0.000	0.000
LFHF, ms^2^/ms^2^	0.81 (0.36; 1.69)	7.29 (4.84; 12.25)	0.36 (0.25; 0.81)	0.000	0.000	0.000
IC_HR_, ms^2^/ms^2^	1.16 (0.53; 2.42)	7.63 (5.12; 12.99)	0.94 (0.51; 1.70)	0.000	0.002	0.000

Abbreviations: TP, dispersion of RR, intervals in a given time interval ≈≤ 0.4 hz; (total power); VLF, power spectrum of HRV, in the very-low frequency range ≤0.04 Hz; LF, power spectrum of HRV, in the low frequency range 0.04–0.15 Hz; LFn, LF/(TP–VLF)×100; spectrum power of HRV, in the low-frequency range in normalized units; HF, power spectrum of HRV, in the high frequency range 0.15–0.4 hz; HFn, HF/(TP–VLF)×100; spectrum power of HRV, in the high-frequency range in normalized units; LFHF, LF[ms^2^]/HF[ms^2^]; IC_HR_, (LF[ms^2^]+ VLF[ms^2^])/HF[ms^2^]; centralization index of heart rate.

That is, it can be argued that the diagnosis of the functional state of the athletes’ body based on the analysis of point recordings of HRV during spontaneous breathing without taking into account RR is insufficiently informative on the one hand, and on the other hand, it depends on the pattern of forced breathing, which can be formed due to external (psycho-emotional and physical exertion, oxygen content in the air, etc.), internal factors (of processes of ensuring external, hemic and cellular respirations, water-salt and endocrine homeostasis, etc.), as well as the possibilities of management for it. Currently, there are a huge number of publications that characterize changes in HRV in various states, but the vast majorities of them considers these changes without taking into account indicators of frequency, depth, and pattern breathing, and are limited to characterizing changes in HRV indicators in various states and under various influences.

SDNN, RMSSD, and pNN50 calculated from an array of cardio intervals (R-R intervals) are usually used as statistical indicators. SDNN reflects the balance of sympathetic and parasympathetic influences on SR. RMSSD and pNN50 indicators are associated with parasympathetic influences. Geometric methods of analysis are based on the calculation of the mode of the distribution of cardio intervals and the amplitude of the mode. On the basis of geometric indicators of HRV, Bayevsky indicators are calculated - SI (c.u.), ABI (c.u.), SRAI (c.u.), ARI (c.u.) (Bayevsky, 2004). SI (c.u.) is most often used - a stress index that reflects the degree of predominance of the activity of central regulatory mechanisms over autonomous ones. Considering the data presented in Table 7, it can be stated that most of the indicators of statistical and geometric analysis of HRV in terms of the direction and orientation of shifts when performing a breathing maneuver differ from the initial state. The latter confirms their validity in differentiating the activation of the sympathetic and parasympathetic branches of the ANS. The exception is the lower sensitivity of the ABI and RMSSD indicators to the activation of sympathetic influences during hyperventilation and the pNN50 indicator to the suppression of the activity of sympathetic influences during CR_6_ (Table 7).

**TABLE 7:** Indicators of statistical and geometric analysis of HRV of the examined athletes during the breathing maneuver, Med (Q_1_; Q_3_), n = 183.

Indicator	SR	CR_6_	CR_15_	p		
				SR-CR_6_	SR-CR_15_	CR_6_-CR_15_
ABI, c.u	14.77 (9.65; 28.15)	6.10 (4.01; 10.57)	17.96 (9.83; 31.04)	0.000	0.145	0.000
SRAI, c.u	4.06 (2.90; 5.45)	2.71 (2.12; 3.60)	5.01 (3.55; 7.23)	0.000	0.000	0.000
ARI, c.u	4.75 (3.29; 7.68)	3.57 (2.61; 5.29)	5.87 (4.06; 8.91)	0.000	0.007	0.000
SI, c.u	84.10 (47.05; 162.27)	39.24 (27.20; 64.41)	119.59 (63.03; 206.78)	0.000	0.001	0.000
SDANN, ms	61.82 (48.34; 83.49)	130.81 (106.84; 152.11)	51.95 (40.93; 69.00)	0.000	0.000	0.000
RMSSD, ms	48.0 (33.5; 69.9)	67.7 (48.3; 93.4)	47.6 (33.2; 74.4)	0.000	0.470	0.000
pNN50, %	12.5 (10.9; 14.9)	12.5 (10.8; 22.7)	11.8 (9.7; 18.0)	0.813	0.000	0.000

Abbreviations: ABI, autonomic balance index; SRAI, subcortical regulation adequacy indicator; ARI, autonomic regulation index; SI, stress index; SDANN, standard deviation of the values of cardiointervals; RMSSD, square root of the sum of squares of the differences in the values of consecutive pairs of normal intervals; pNN50, the percentage of NN50 from the total number of consecutive pairs of intervals that differ by more than 50 milliseconds, obtained over the entire time recording.

However, even such generally accepted indicators of geometric and statistical analysis as SI (c.u.), RMSSD and pNN50 change significantly and vary within the diagnostic limits of different conditions when RR changes. As an example, the SI indicator (c.u.), varying in the range from 39.24 to 119.59, can characterize the optimal state in the first case and the state of pronounced tension of heart rhythm regulation in the second, which casts significant doubt on the validity of such conclusions.

### 3.4 Breathing maneuver and blood pressure variability

In Table 8 shows the average values of SBPf, DBPf and PBPf, measured on the finger using the Penaz method. The method is quite sensitive to the body`s position, the location of the finger, its preparation, movements, so the measured indicators often differ from those measured using the shoulder cuff. However, meticulous preparation of the measurement site in a sitting position allowed avoiding these shortcomings. In more modern models of this device, the problem was solved (Pivovarov et al., 2015). When performing a respiratory maneuver, SBPf at CR_6_ and CR_15_ significantly decreases compared to SR, *p* = 0.000, although it does not differ between them, *p* = 0.628. That is, both breathing options cause a decrease in SBP. Similarly, DBPf changes. PBPf also decreases in both cases. In this case, we will limit ourselves to stating the results of the measurement of these indicators.

**TABLE 8:** Changes in the average values of blood pressure from the finger cuff in the subjects of the examined athletes during the breathing maneuver, Med (Q_1_; Q_3_), n = 183.

Indicator	SR	CR_6_	CR_15_	p		
				SR-CR_6_	SR-CR_15_	CR_6_-CR_15_
SBPf, mmHg	112.5 (110.0; 120.0)	110.2 (104.0; 120.2)	111.1 (102.3; 120.8)	0.000	0.000	0.628
DBPf, mmHg	66.9 (52.4; 77.9)	66.2 (45.4; 76.1)	63.6 (48.1; 75.4)	0.000	0.000	0.750
PBPf, mmHg	48.4 (41.2; 63.3)	44.1 (37.0; 60.9)	43.9 (36.8; 60.9)	0.013	0.011	0.764

Abbreviations: SBPf, systolic blood pressure (from finger by Penaz); DBPf, diastolic blood presure (from finger by Penaz); PBPf, pulse blood pressure (from finger by Penaz).

The issue of assessing blood pressure variability (BPV) is more difficult. In the literature, this concept almost always refers to changes in one-time BP values (often performed by auscultatory method) over long periods of time: during a month, a week, a day, during an appointment with a doctor (Parati et al., 2021). BPV assessment in beet-to-beet mode is only available if continuous BP recording is technically possible, and is therefore not as common. However, a number of works on its analysis have appeared in recent years (Rosei et al., 2020). There are certain shortcomings of the physiological justification of BP variability parameters. Available publications concern SBP exclusively (Cottin et al., 2008; Abreu. et al., 2022; Volpes et al., 2022). In our and many other studies, the relationship of the total power of SBP variability (ТР_SBP_, mmHg^2^) with the value of SBP (mmHg) was shown, which is definitely a negative prognostic factor when higher values are reached (Parati and Valentini, 2006). This indicator is actively used to predict the severity of cardiac arrhythmias (Mainardi et al., 2009; Corino et al., 2014; Feenstra et al., 2018). There are publications that connect HF_SBP_ with breathing and prove its connection with the level of endothelial NO activity (Lacchini et al., 2001; Gamboa et al., 2007), VLF_SBP_ with the activity of the angiotensin-renin system (Cheng et al., 2010). There is evidence that LF_SBP_ reflects sympathetic and myogenic modulation of vascular tone (Langager et al., 2007). According to modern ideas, based on neuroimaging data, there is a morphofunctional formation in the brain, the so-called “sympathetic connectome”, which determines suprabulbar influences on BP (Macefield and Henderson, 2019), whose influences are also correlated with the magnitude of LF_SBP_. Considering the significant number of factors that influence SBP, the main thing in the interpretation of these indicators is a general understanding of the impact on the pumping function of the heart, which must be adjusted to different physiological and pathological conditions (Romanchuk, 2018; Guzii and Romanchuk, 2021a). Although clinicians try to highlight the special physiological meaning of each of the components of SBP variability. Regarding the variability of DBP, it is accepted that DBP largely reflects the resistance of the arterial wall (Corino et al., 2014; Cherepov, 2015; Kishi, 2018), although there are almost no publications regarding DBP variability.

We can be seen from Table 9 executions of the respiratory maneuver leads to a number of changes in blood pressure variabiliy indicators. Most of them are significant. First of all, it is advisable to focus on indicators that did not change during the performance of this test, or changed specifically. The increase in VLF_SBP_, VLF_DBP_ and HF_DBP_ at CR_6_ and at CR_15_ was of the same type and did not differ (Table 9), which probably indicates common mechanisms of response to controlled breathing. After all, the study of changes in these indicators under the influence of intense training load showed their stable changes during the formation of overstrain of the sympathetic type (Guzii et al., 2021). There was a significant difference in HF_SBP_ at CR_15_. Resonantly to RR, there is an increase in LF_SBP_ at CR_6_, which resembles an increase in LF (Table 9). On the other hand, at CR_15_, LF_SBP_ returns to initial values, in contrast to LF (Table 6) and LF_DBP_ (Table 9), which decrease below initial values. That is, it can be assumed that when performing a breathing maneuver, which first suppresses the activation of sympathetic mechanisms (CR_6_), and then activates them (CR_15_), a certain dissociation of sympathetic effects on the heart rhythm, contractile function of the heart, and vascular tone occurs. At the same time, the increase in LF effects on the mentioned functions at CR_6_ is more resonant. Taking into account the indicators of the total spectral power of BPV (TP_SBP_, TP_DBP_), it can be argued that inhibition of activation of the postganglionic sympathetic nerve and sympathoadrenal activation due to hyperventilation against the background of activation of the suction function of the chest at CR_6_ and CR_15_ engages more mechanisms of SBP support than DBP. It is likely that deep liquid breathing (CR_6_) causes greater synchronization of arterial tone changes than more frequent deep breathing (CR_15_).

**TABLE 9:** Changes in SBP and DBP variability indicators of the examined athletes during the breathing maneuver, Med (Q_1_; Q_3_), n = 183.

Indicator	SR	CR_6_	CR_15_	p		
				SR-CR_6_	SR-CR_15_	CR_6_-CR_15_
TP_SBP_, mmHg^2^	26.0 (16.0; 43.6)	64.0 (39.7; 94.1)	38.4 (23.0; 64.0)	0.000	0.000	0.000
VLF_SBP_, mmHg^2^	10.2 (4.4; 21.2)	13.7 (6.3; 27.0)	13.0 (6.3; 29.2)	0.007	0.005	0.856
LF_SBP_, mmHg^2^	6.8 (4.0; 10.9)	36.0 (23.0; 57.8)	6.3 (3.2; 11.6)	0.000	0.629	0.000
LF_SBP_n,n.u	52.9 (35.8; 68.8)	88.9 (80.3; 93.6)	31.1 (18.6; 45.2)	0.000	0.000	0.000
HF_SBP_, mmHg^2^	4.8 (2.9; 9.6)	4.0 (2.3; 6.8)	12.3 (7.3; 24.0)	0.001	0.000	0.000
HF_SBP_n,n.u	44.4 (29.2; 61.5)	9.7 (5.9; 16.9)	64.7 (52.1; 77.9)	0.000	0.000	0.000
LFHF_SBP_, mmHg^2^/mmHg^2^	1.19 (0.59; 2.34)	9.18 (4.79; 15.92)	0.48 (0.23; 0.88)	0.000	0.000	0.000
TP_DBP_, mmHg^2^	10.2 (6.8; 16.0)	24.0 (15.2; 36.0)	10.9 (6.8; 19.4)	0.000	0.272	0.000
VLF_DBP_, mmHg^2^	3.6 (2.0; 6.3)	4.8 (2.6; 9.0)	4.4 (2.3; 7.3)	0.001	0.008	0.585
LF_DBP_, mmHg^2^	4.0 (2.3; 6.8)	14.4 (7.8; 23.0)	2.6 (1.7; 4.4)	0.000	0.000	0.000
LF_DBP_n,n.u	74.4 (59.3; 81.7)	85.6 (78.3; 91.2)	57.3 (37.7; 74.9)	0.000	0.000	0.000
HF_DBP_, mmHg^2^	1.2 (0.6; 2.0)	2.0 (1.2; 3.2)	1.7 (1.0; 3.6)	0.000	0.000	0.978
HF_DBP_n,n.u	22.5 (15.5; 35.3)	12.5 (8.0; 19.3)	37.1 (22.8; 56.8)	0.000	0.000	0.000
LFHF_DBP_, mmHg^2^/mmHg^2^	3.35 (1.66; 5.34)	6.81 (4.08; 11.29)	1.54 (0.69; 3.28)	0.000	0.000	0.000
IC_SBP_, mmHg^2^/mmHg^2^	3.71 (1.36; 8.21)	13.30 (7.84; 25.04)	1.57 (0.80; 3.09)	0.000	0.000	0.000
IC_DBP_, mmHg^2^/mmHg^2^	7.36 (4.20; 12.72)	10.40 (5.81; 17.84)	4.30 (2.01; 9.18)	0.000	0.000	0.000
BR_LF_, ms×mmHg^-1^	14.2 (9.7; 20.1)	20.4 (14.6; 26.7)	9.4 (6.8; 12.9)	0.000	0.000	0.000
BR_HF_, ms×mmHg^-1^	18.3 (11.9; 27.5)	20.8 (15.4; 30.4)	10.3 (6.2; 15.6)	0.000	0.000	0.000

Abbreviations: TP_SBP_, dispersion of SBPf, in a given time interval (total power) ≈≤ 0.4 hz; VLF_SBP_, power spectrum of SBPf, in the very-low frequency range ≤0.04 Hz; LF_SBP_, power spectrum of SBPf, in the low frequency range 0.04–0.15 Hz; LF_SBP_n, LF_SBP_/(TP_SBP_–VLF_SBP_)×100; spectrum power of SBPf, in the low-frequency range in normalized units; HF_SBP_, power spectrum of SBPf, in the high frequency range 0.15–0.4 hz; HF_SBP_n, HF_SBP_/(TP_SBP_–VLF_SBP_)×100; spectrum power of SBPf, in the high-frequency range in normalized units; LFHF_SBP_, LF_SBP_[mmHg^2^]/HF_SBP_[mmHg^2^]; TP_DBP_, dispersion of DBPf, in a given time interval (total power) ≈≤ 0.4 hz; VLF_DBP_, power spectrum of DBPf, in the very-low frequency range ≤0.04 Hz; LF_DBP_, power spectrum of DBPf, in the low frequency range 0.04–0.15 Hz; LF_DBP_n, LF_DBP_/(TP_DBP_–VLF_DBP_)×100; spectrum power of DBPf, in the low-frequency range in normalized units; HF_DBP_, power spectrum of DBPf, in the high frequency range 0.15–0.4 hz; HF_DBP_n, HF_DBP_/(TP_DBP_–VLF_DBP_)×100; spectrum power of DBPf, in the high-frequency range in normalized units; LFHF_SBP_, LF_SBP_[mmHg^2^]/HF_SBP_[mmHg^2^]; IC_SBP_, (LF_SBP_[mmHg^2^]+ VLF_SBP_[mmHg^2^])/HF_SBP_ [mmHg^2^]; centralization index of systolic blood pressure; IC_DBP_, (LF_DBP_[mmHg^2^]+ VLF_DBP_[mmHg^2^])/HF_DBP_[mmHg^2^]; centralization index of diastolic blood pressure; BR_LF,_ sensitivity of arterial baroreflex in low frequency range (√LF [ms^2^]×LF[mmHg^2^]^−1^); BR_HF_, sensitivity of arterial baroreflex in high frequency range (√HF [ms^2^]×HF[mmHg^2^]^−1^).

That is, in view of the development of possible pre-pathological conditions during sports, changes in blood pressure variability indicators, which determine of the circulatory system reaction to controlled breathing, under the condition of an insignificant or excessive reaction (for example, TP_SBP_, TP_DBP_, LF_SBP_ and LF_DBP_), or appearing of reaction (as an example, VLF_SBP_, VLF_DBP_ and HF_DBP_) are important. The latter may characterize various mechanisms of dysregulation, which may be associated with neuroendocrine processes that occur in the athlete’s body during the training cycle.

Indices of centralization for blood pressure (IC_SBP_ and IC_DBP_) were calculated similarly to HR. At SR and CR_15_, a significant predominance of central influences on DBP is noted in terms of absolute values, and at CR_6_ on SBP.

Indicators of baroreflex sensitivity (BR_LF_ and BR_HF_) are keys in determining mechanisms for maintaining vascular homeostasis and indicators of autonomic control (Kawada et al., 2014). According to other authors, baroreflex sensitivity is also important in ensuring cerebral blood circulation (Ogoh and Tarumi, 2019). The quantitative value that expresses its effectiveness is the value that is obtained with the dimension ms×mmHg^-1^. Two well-known methods are implemented in the SAСR device (Robbe. et al., 1987; Parati, 2005; Rydlewska et al., 2010). We used the spectral method of determining the sensitivity of the arterial baroreflex. From the results presented in Table 9 shows that the sensitivity of the arterial baroreflex significantly increases with deep slow breathing, more significantly in the HF range. From 14.2 (9.7; 20.1) to 20.4 (14.6; 26.7), *p* = 0.000 for BR_LF_ (ms×mmHg^-1^) and from 18.3 (11.9; 27.5) to 20.8 (15.4; 30.4), *p* = 0.000 for BR_HF_ (ms ×mmHg^-1^). At the same time, with deep, more frequent breathing (CR_15_), it significantly decreases, even in comparison with SR to 9.4 (6.8; 12.9), *p* = 0.000 for BR_LF_ (ms×mmHg^-1^) and to 10.3 (6.2; 15.6), *p* = 0.000 for BR_HF_ (ms×mmHg^-1^). The effects of decreased baroreflex sensitivity are usually associated with a decrease in the LF component of HRV. They were also obtained by us under the influence of training and competition loads, when the LF component of HRV decreases against the background of an increase in HR, but then the effect of an increase in the LF component of SBP is also noted (Guzii and Romanchuk, 2016), which further affects the baroreflex sensitivity indicator. In general, the obtained results confirm the known data on the influence of controlled breathing on the sensitivity of the baroreflex (Radaelii et al., 2004; Parati, 2005; Li et al., 2018; Incognito et al., 2019; Lukarski et al., 2022). For the unification of studies, they are conducted at a breathing frequency of 0.1 Hz. In this case, the likely search for differences may relate to the features of BR_LF_ (ms×mmHg^-1^) and BR_HF_ (ms×mmHg^-1^) changes in different breathing modes under the influence of various factors, which may have prognostic value (Wang et al., 2013), including in relation to functional and non-functional overreaching of athletes.

### 3.5 Breathing maneuver and indicators of hemodynamics

Determining hemodynamic indicators is an important research method for determining the state of blood circulation, diagnosing diseases of the cardiovascular system, and predicting the adaptive capabilities of the body (DeBoer et al., 1987). Key indicators are cardiac stroke volume, cardiac output, peripheral vascular resistance, end-diastolic and end-systolic volumes, as well as ejection fraction. Rheographic and ultrasound methods are more often used when working with healthy people (Galasko et al., 2004; García et al., 2011). Impedance (Kubicek et al., 1966; Scherhag et al., 2005) and inductive cardiography (Kaplan et al., 2003) are also used. This approach found its continuation in new methods of non-invasive determination of SV based on continuous BP registration by Finometer-type devices (Headley, 2006; Reisner et al., 2011). The Modelflow method implemented in the Finometer calculates the aortic flow waveform based on peripheral arterial pressure by simulating a nonlinear three-element model of aortic input resistance. The methodology tracks rapid changes in stroke volume during various experimental protocols, including postural stress and exercise. However, if absolute values are required, a gold standard calibration is required (Bogert and van Lieshout, 2005; Gibbons et al., 2019). Another approach is the calculation of SV by the method of two-phase reconstruction based on the parameters of the averaged cardiac complex - by electrical systole. The last algorithm is used in the SACR device. The method showed a high degree of correlation with methods of multiphase reconstruction. It provides a clear definition of the intervals of the PQRST complex (Kim et al., 2005). This possibility exists when determining the average PQRST complex after registration, which can be adjusted.

**FIGURE 5:**
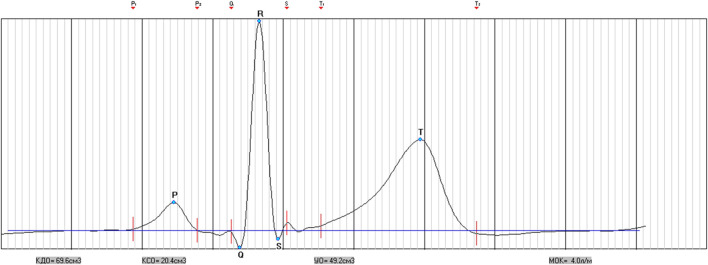
Cross-section complex PQRST obtained during SACR registration.

That is, a feature of this approach is the discrete determination of hemodynamic indicators for a certain period of time (registration time). Therefore, receiving separate indicators, we can talk about how they changed during this or that component of the breathing maneuver. Of course, the wave changes that are registered at each heart contraction in the aorta allow to reveal the direct variability of SV (cm^3^) by the resistance difference in the aorta, which is achieved using the Modelflow method (Bogert and van Lieshout, 2005). However, the issue of quantitative assessment of the volume and its comparability remains not fully resolved. On the other hand, it is possible to analyze not the average, but the direct complex PQRST (Figure 5).

As can be seen from Table 10 parameters of hemodynamics obtained from the results of the analysis of cross-sectional PQRST complexes indicate changes during the performance of individual parts of the respiratory maneuver. A gradual significant decrease in SV (cm^3^) from 66.0 (60.3; 73.9) at SR to 65.7 (60.1; 72.9) at CR_6_, *p* = 0.010 and to 63.6 (57.9; 71.2) at CR_15_, *p* = 0.000, is accompanied by a significant increase in ESV (cm^3^) at CR_6_, *p* = 0.022, which at CR_15_ does not differ from either SR or CR_6_. At the same time, EDV (cm^3^) at CR_6_ does not differ from SR, 96.6 (85.2; 109.0) *versus* 95.9 (85.2; 109.2), *p* = 0.741, and at CR_15_ it significantly decreases to 94.4 (84.9; 105.3), *p* = 0.000. During the respiratory maneuver, there is a significant increase in CO (dm^3^) from 4.5 (4.1; 5.0) at SR to 4.8 (4.3; 5.2) at CR_6_, *p* = 0.000, to 5.0 (4.5; 5.5) at CR_15_, *p* = 0.000. The opposite dynamics is observed from the side of GPVR (dyn/s/cm^−5^), which significantly decreases during the breathing maneuver from 1,577 (1,409; 1740) at SR to 1,417 (1,288; 1,585) at CR_15_, *p* = 0.000. It is likely that an increase in the preload on the heart during CR_6_ increases the force of contraction, but does not increase EDV (cm^3^), but is compensated by an increase during the maneuver HR (min^-1^), *p* = 0.000 (Table 3), a decrease in SBPf (mmHg), *p* = 0.000 and a certain tension of the contractile function of the heart due to an increase in QTc (s). At CR_15_, EDV (cm^3^) decreases probably due to the fact that there is a further increase in HR (min^-1^), which is associated with hyperventilation, as well as more stress on the contractile function of the heart.

**TABLE 10:** Changes in the hemodynamic parameters of the examined athletes during the breathing maneuver, Med (Q_1_; Q_3_), n = 183.

Indicator	SR	CR_6_	CR_15_	p		
				SR-CR_6_	SR-CR_15_	CR_6_-CR_15_
EDV, cm^3^	95.9 (85.2; 109.2)	96.6 (85.2; 109.0)	94.4 (84.9; 105.3)	0.741	0.000	0.000
ESV, cm^3^	29.7 (23.5; 37.3)	30.6 (24.4; 36.1)	30.6 (24.1; 35.6)	0.022	0.156	0.577
SV, cm^3^	66.0 (60.3; 73.9)	65.7 (60.1; 72.9)	63.6 (57.9; 71.2)	0.010	0.000	0.000
CO, dm^3^	4.5 (4.1; 5.0)	4.8 (4.3; 5.2)	5.0 (4.5; 5.5)	0.000	0.000	0.000
CI, dm^3^×m^-2^	2.39 (2.16; 2.62)	2.50 (2.26; 2.78)	2.59 (2.33; 2.94)	0.000	0.000	0.000
GPVR, dyn/s/cm^−5^	1,577 (1,409; 1740)	1,486 (1,363; 1,628)	1,417 (1,288; 1,585)	0.000	0.000	0.000
SI, cm^3^×m^-2^	34.9 (31.1; 38.8)	34.3 (31.2; 38.2)	33.2 (29.9; 37.2)	0.017	0.000	0.000

Abbreviations: EDV, end-diastolic volume; ESV, end-systolic volume; SV, stroke volume; CO, cardiac output; CI, cardiac index; GPVR, general periferical vascular resistance; SI, stroke index.

That is, the respiratory maneuver, in our opinion, can characterize the features and ability of the hemodynamic system to adapt to changing conditions. In this case is of a minor nature. However, further research into the possibility of its use is necessary.

### 3.6 Breathing maneuver and indicators of the cardiorespiratory system synchronization

The question of synchronizing the work of the cardiovascular and respiratory systems is one of the key issues of revealing the mechanisms of their interaction in healthy, physically fit people, as well as patients at various stages of the development of the pathological process, convalescence and rehabilitation. According to many scientists, the search for solutions lies in the plane of reflection and interaction of time and wave processes, characteristic of many physiological systems that ensure the activity of the human body (Kapidžić et al., 2014; Örün et al., 2021; Kalauzi et al., 2023). There are a number of internal and external factors (Schulz et al., 2013; Zhao et al., 2019) that make it impossible to unambiguously solve this issue. However, the search for simple, understandable indicators for practical application also continues (Noskin et al., 2018; Noskin et al., 2020; Matić et al., 2022). After all, the main application of knowledge regarding the synchronization of the activities of the respiratory and cardiovascular systems, in our opinion, consists in the development of methods for correcting the functional state of the body through the use of methods of voluntary control of breathing, taking into account the frequency, respiratory volume and rhythm of breathing (Vaschillo et al., 2006; Gross et al., 2017; Skytioti and Elstad, 2022). The latter, by the way, is important both in the practice of sports medicine and in the practice of rehabilitation after various injuries and diseases (Zoccal et al., 2022). It should also be added the significant role of breathing control in the correction of psycho-emotional states (Krohn et al., 2023).

A well-known and widely used indicator that reflects cardiorespiratory interaction is the Hildebrandt index (Hildebrandt, 1953). However, almost 70 years of its use could not provide convincing information about its informativeness. There are only certain assumptions about the possibility of characterizing the state of the autonomic nervous system with its help. On the other hand, simultaneous measurement of HR and RR parameters before the introduction of cardiopulmonary testing was rarely carried out, and when it was carried out, it was often a subjective method. Currently available simultaneous measurement technologies are able to supplement the known data taking into account many components that affect the value of this indicator. Currently, technologies for clothing and devices are being developed, which will allow more accurate determination and clear analysis of this parameter in various conditions. As an example, we can cite the data obtained by us during the manual correction of disorders in the thoracic spine, when this indicator changed due to significant changes in the duration of exhalation after the correction of the spine (Romanchuk, 2022a; Romanchuk, 2022b). The technology of simultaneous measurement of indicators of the cardiovascular and respiratory systems makes it possible to develop a number of both frequency and discrete parameters of cardiorespiratory interaction (Noskin et al., 2018; Noskin et al., 2020; Matić et al., 2022).

The simultaneous recording of ECG and inspiratory flow allowed us to test for use a discrete parameter that characterizes the relationship between cardiac output and minute ventilation (VSI). It takes into account the adaptive changes of both the cardiovascular and respiratory systems, which are somehow related to each other, due to the entry and transport of oxygen in the body.

Quite expected (Table 11) are changes in the HI indicator, which are associated with RR and at CR_15_ do not significantly, differ from SR, unlike CR_6_. VSI is inversely related to V (L×min^-1^), which is largest at CR_15_, so VSI is smallest. As for the dynamic changes of this indicator, it can be argued that its significant decrease is a negative criterion for the activity of the cardiovascular system, and an increase, as a rule, will indicate the economization of the function.

**TABLE 11:** Changes in indicators of synchronization of cardiorespiratory interaction in the examined athletes during the breathing maneuver, Med (Q_1_; Q_3_), n = 183.

Indicator	SR	CR_6_	CR_15_	p		
				SR-CR_6_	SR-CR_15_	CR_6_-CR_15_
VSI, dm^3^× L^-1^	0.586 (0.477; 0.745)	0.441 (0.327; 0.582)	0.360 (0.247; 0.477)	0.000	0.000	0.000
Hildebrandt index	4.77 (3.98; 6.20)	10.92 (9.97; 12.14)	5.00 (4.48; 5.84)	0.000	0.209	0.000

Abbreviations: VSI, CO (dm^3^)/V (L×min^-1^); volume synchronization index; Hildebrandt index, HR (min^-1^)/RR (min^-1^), rate synchronization index.

Summarizing the obtained results, the increases in the indicators of the cardiorespiratory system during the breathing maneuver (Table 12) were worked out, which proved the ranges of expected (within Q1 - Q3) changes, and also indicated the limits of changes that can characterize the inadequacy of the response of the cardiorespiratory system. The latter may be caused by changes in the body of athletes that increase resistance to stimulation (when breathing at 0.25 Hz) or suppression (when breathing at 0.1 Hz) of sympathoadrenal influences.

**TABLE 12:** Increments of indicators in the examined athletes during the breathing maneuver at CR_6_ and CR_15_ compared to SR, Med (Q_1_; Q_3_), n = 183.

	δ SR—CR_6_	δ SR—CR_15_	z	p
δ HR, min^-1^	2.3 (0.2; 5.9)	8.1 (4.3; 13.4)	11.3	0.000
δ QTc, s	0.004 (−0.002; 0.010)	0.013 (0.005; 0.019)	9.9	0.000
δ ST,n.u	0.008 (−0.014; 0.030)	0.005 (−0.027; 0.032)	0.5	0.652
δ TP,ms^2^	13,918.5 (7,681.4; 19,471.6)	−1,003.2 (−3,419.4; 327.4)	11.7	0.000
δ VLF,ms^2^	146.7 (−230.7; 585.6)	51.1 (−330.3; 456.0)	1.5	0.142
δ LF,ms^2^	13,683.4 (8,402.0; 18,107.7)	−656.5 (−1762.6; −108.0)	11.7	0.000
δ LFn,n.u	44.3 (23.6; 60.6)	−8.7 (−26.8; 1.4)	11.7	0.000
δ HF,ms^2^	26.7 (−1,019.2; 1,437.2)	−216.6 (−1,156.3; 428.0)	4.4	0.000
δ HFn,n.u	−42.7 (−59.5; −22.4)	8.6 (−2.6; 26.0)	11.7	0.000
δ LFHF,ms^2^/ms^2^	5.89 (3.60; 10.12)	−0.20 (−1.19; 0.00)	11.7	0.000
δ TP_SBP_, mmHg^2^	29.7 (9.0; 58.6)	9.8 (−6.2; 31.2)	6.4	0.000
δ TP_DBP_, mmHg^2^	13.0 (4.6; 23.4)	0.9 (−5.0; 5.9)	9.7	0.000
δ VLF_SBP_, mmHg^2^	3.0 (−6.5; 13.4)	2.8 (−5.8; 15.1)	0.2	0.855
δ VLF_DBP_, mmHg^2^	1.0 (−1.4; 3.7)	1.0 (−1.8; 4.0)	0.5	0.584
δ LF_SBP_, mmHg^2^	28.4 (13.6; 49.0)	−0.3 (−4.4; 4.0)	11.2	0.000
δ LF_DBP_, mmHg^2^	9.7 (4.6; 16.8)	−0.6 (−2.9; 0.6)	11.5	0.000
δ LF_SBP_n, n.u	34.1 (13.8; 50.4)	−18.3 (−34.1; −2.2)	11.7	0.000
δ LF_DBP_n, n.u	11.1 (3.0; 23.4)	−13.3 (−28.1; −1.5)	11.5	0.000
δ HF_SBP_, mmHg^2^	−0.8 (−4.6; 1.3)	6.2 (1.4; 16.1)	10.6	0.000
δ HF_DBP_, mmHg^2^	0.7 (0.0; 1.9)	0.4 (−0.3; 2.0)	0.03	0.976
δ HF_SBP_n, n.u	−32.2 (−49.0; −11.5)	18.5 (2.4; 32.7)	11.7	0.000
δ HF_DBP_n, n.u	−8.3 (−20.9; −2.4)	11.6 (0.8; 26.0)	11.4	0.000
δ LFHF_SBP_, mmHg^2^/mmHg^2^	6.72 (2.77; 13.65)	−0.49 (−1.50; −0.07)	11.7	0.000
δ LFHF_DBP_, mmHg^2^/mmHg^2^	3.24 (0.86; 7.07)	−1.28 (−2.82; −0.09)	11.3	0.000
δ TP_R_, (L×min^-1^)^2^	256.1 (45.3; 711.5)	710.4 (194.4; 1,684.6)	9.9	0.000
δ VLF_R_, (L×min^-1^)^2^	7.0 (3.2; 15.4)	2.2 (0.0; 6.6)	7.8	0.000
δ LF_R_, (L×min^-1^)^2^	478.7 (215.8; 972.2)	2.2 (−24.5; 18.2)	11.7	0.000
δ LF_R_, n, n.u	75.0 (59.1; 82.9)	−2.5 (−14.1; −0.7)	11.7	0.000
δ HF_R_, (L×min^-1^)^2^	−185.1 (−326.3; −74.9)	722.4 (219.5; 1744.9)	11.7	0.000
δ HF_R_, n, n.u	−70.5 (−79.7; −53.0)	7.4 (2.1; 20.0)	11.7	0.000
δ LFHF_R_, (L×min^-1^)^2^/(L×min^-1^)^2^	6.11 (3.48; 8.29)	−0.03 (−0.18; −0.01)	11.7	0.000
δ T_I_,s	2.5 (1.7; 2.9)	−0.0 (−0.4; 0.3)	11.7	0.000
δ T_E_,s	2.9 (2.3; 3.6)	−0.3 (−0.9; 0.1)	11.7	0.000
δ V_T_, L	1.10 (0.70; 1.69)	0.32 (0.12; 0.68)	11.1	0.000
δ V_T_/T_I_, L×s^-1^	0.09 (0.02; 0.17)	0.20 (0.10; 0.36)	8.6	0.000
δ V_T_/T_E_, L×s^-1^	0.08 (−0.01; 0.22)	0.21 (0.08; 0.37)	10.1	0.000
δ Ti/(Ti + Te), c.u	0.03 (−0.02; 0.07)	0.03 (−0.01; 0.07)	2.5	0.011
δ RR, min^-1^	−7.7 (−10.3; −5.5)	1.1 (−1.5; 3.4)	11.7	0.000
δ V, L×min^-1^	3.0 (0.9; 6.2)	6.1 (3.0; 10.4)	9.2	0.000
δ VO_2_	0.14 (0.04; 0.28)	0.28 (0.14; 0.48)	9.2	0.000
δ EDV, cm^3^	0.0 (−3.8; 3.5)	−1.8 (−6.4; 1.8)	4.3	0.000
δ ESV, cm^3^	0.4 (−1.2; 2.4)	0.4 (−1.7; 2.3)	0.6	0.577
δ SV, cm^3^	−0.6 (−2.8; 1.7)	−2.2 (−5.3; 0.1)	6.7	0.000
δ CO, dm^3^	0.2 (0.0; 0.4)	0.4 (0.1; 0.7)	7.0	0.000
δ CI, dm^3^×m^-2^	0.103 (0.000; 0.203)	0.206 (0.059; 0.353)	7.0	0.000
δ GPVR, dyn/s/cm^−5^	−78.6 (−170.4; 6.5)	−131.0 (−262.3; −32.2)	6.0	0.000
δ SI, cm^3^×m^-2^	−0.22 (−1.49; 0.89)	−1.12 (−2.85; 0.10)	6.4	0.000
δ VSI, dm× L ^-1^	−0.128 (−0.255; −0.051)	−0.229 (−0.358; −0.128)	8.1	0.000
δ Hildebrandt index	6.02 (4.98; 7.26)	0.28 (−0.82; 1.08)	11.7	0.000
δ BR_LF_, ms×mmHg^-1^	5.05 (0.57; 10.15)	−4.32 (−9.11; −1.52)	11.6	0.000
δ BR_HF_, ms×mmHg^-1^	3.31 (−3.89; 9.20)	−6.91 (−11.86; −2.82)	10.5	0.000
δ IC_HRV_, ms^2^/ms^2^	5.98 (3.37; 10.92)	−0.13 (−1.11; 0.41)	11.7	0.000
δ IC_SBP_, mmHg^2^/mmHg^2^	8.18 (2.69; 19.93)	−1.52 (−6.36; 0.10)	11.2	0.000
δ IC_DBP_, mmHg^2^/mmHg^2^	2.04 (−2.23; 7.74)	−2.44 (−6.65; 1.03)	8.0	0.000
δ IC_R_, (L×min^-1^)^2^/(L×min^-1^)^2^	6.20 (3.58; 8.46)	−0.04 (−0.22; −0.01)	11.7	0.000
δ ABI, c.u	−7.74 (−18.16; −1.70)	0.74 (−6.85; 11.94)	9.9	0.000
δ SRAI, c.u	−1.16 (−2.08; −0.39)	1.06 (0.03; 2.11)	11.4	0.000
δ ARI, c.u	−1.17 (−3.45; 0.26)	0.51 (−1.18; 2.37)	8.0	0.000
δ SI, c.u	−37.67 (−105.23; −7.31)	14.52 (−26.51; 90.98)	10.4	0.000
δ SDANN, ms	59.53 (41.02; 79.39)	−10.47 (−24.62; 2.05)	11.7	0.000
δ RMSSD, ms	13.63 (0.26; 35.57)	−0.43 (−13.94; 19.37)	5.9	0.000
δ pNN50, %	−0.26 (−1.29; 7.80)	−0.94 (−2.35; −0.13)	5.5	0.000

In this work, we will consider some variants of changes that are unlikely, and when they appear, we can speak with a significant degree of probability about the formation of states of the organism with high or low resistance to the proposed breathing maneuver. Some of these conditions may indicate a high level of functional status, while others may indicate a significant decrease. First of all, we will describe changes in the well-known indicators of HRV and arterial baroreflex, which are widely used in the practice of sports medicine and rehabilitation in order to determine the conditions and reactions of the athlete’s or patient’s body.

Given the peculiarities of the percentile statistical distribution, beyond Q3 and Q1 there are values that are intermediate, relative to the expected, or even less expected (beyond the 1 and 99 percentiles), which may indicate a pathological condition. Let’s stop at a wider range and consider it from the standpoint of performing individual components of the maneuver.

Thus, for the reaction to breathing with a frequency of 0.1 Hz when performing a maneuver, it is less characteristic and shows a reduced reaction:

- increase in δ HR less than 0.2 min^-1^;
- increase in δ TP, less than 7,681.4 (ms^2^);
- increase in δ LF, less than 8,402.0 (ms^2^);
- increase in δ LFn, less than 23.6 (n.u);
- decrease in δ HFn, less than 59.5 (n.u);
- increase in δ LF/HF is less than 3.60 (ms^2^/ms^2^);
- increase in δ Hildebrandt index less than 4.98 (c.u.);
- increase in δ BR_LF_ less than 0.57 (ms×mmHg^-1^);
- decrease in δ BR_HF_ less than 3.89 (ms×mmHg^-1^);
- increase in δ IC_HRV_ is less than 3.37 (ms^2^/ms^2^);
- decrease of δ SRAI less than 2.08 (c.u.);
- decrease of δ SI less than 105.23 (c.u.);
- increase in δ SDANN less than 41.02 (ms);
- increase in δ RMSSD is less than 0.26 (ms);
- decrease in δ pNN50 is less than −1.29 (%).

For the reaction to breathing with a frequency of 0.1 Hz when performing a maneuver, it is less characteristic and indicates an increased reaction:

- increase in δ HR more than 5.9 min-1;
- increase in δ TP, more than 19,471.6 (ms^2^);
- increase in δ LF, more than 18,107.7 (ms^2^);
- increase in δ LFn, more than 60.6 (n.u);
- decrease in δ HFn, more than 22.4 (n.u);
- increase of δ LF/HF more than 10.12 (ms^2^/ms^2^);
- increase in δ Hildebrandt index more than 7.26 (c.u.);
- increase in δ BR_LF_ more than 10.15 (ms×mmHg^-1^);
- decrease of δ BR_HF_ more than 9.20 (ms×mmHg^-1^);
- increase of δ IC_HRV_ more than 10.92 (ms^2^/ms^2^);
- decrease of δ SRAI more than 0.39 (c.u.);
- decrease of δ SI more than 7.31 (c.u.);
- increase of δ SDANN more than 79.39 (ms);
- increase in δ RMSSD more than 35.57 (ms);
- an increase in δ pNN50 of more than 7.80 (%).

For the reaction to breathing with a frequency of 0.25 Hz when performing a maneuver, it is less characteristic and shows a reduced reaction:

- increase in δ HR less than 4.3 min^-1^;
- decrease of δ TP, more than 3,419.4 (ms^2^);
- decrease in δ LF, more than 1762.6 (ms^2^);
- decrease in δ LFn, more than 26.8 (n.u);
- decrease in δ HFn, more than 2.6 (n.u);
- decrease of δ LF/HF more than 1.19 (ms^2^/ms^2^);
- decrease in δ Hildebrandt index more than 0.82 (c.u.);
- decrease of δ BR_LF_ more than 9.11 (ms×mmHg^-1^);
- decrease of δ BR_HF_ more than 11.86 (ms×mmHg^-1^);
- decrease of δ IC_HRV_ more than 1.11 (ms^2^/ms^2^);
- increase in δ SRAI less than 0.03 (c.u.);
- decrease of δ SI more than 26.51 (c.u.);
- decrease of δ SDANN more than 24.62 (ms);
- decrease of δ RMSSD more than 13.94 (ms);
- decrease of δ pNN50 by more than 2.35 (%).

For the reaction to breathing with a frequency of 0.25 Hz when performing a maneuver, it is less characteristic and shows an increased reaction:

- increase in δ HR more than 13.4 min^-1^;
- increase in δ TP, more than 327.4 (ms^2^);
- decrease in δ LF, less than 108.0 (ms^2^);
- increase in δ LFn, more than 1.4 (n.u);
- increase in δ HFn, more than 26.0 (n.u);
- decrease of δ LF/HF less than 0.0 (ms^2^/ms^2^);
- increase in δ Hildebrandt index more than 1.02 (c.u.);
- decrease in δ BR_LF_ less than 1.52 (ms×mmHg^-1^);
- decrease in δ BR_HF_ less than 2.82 (ms×mmHg^-1^);
- increase of δ IC_HRV_ more than 0.41 (ms^2^/ms^2^);
- increase in δ SRAI more than 2.11 (c.u.);
- increase in δ SI more than 90.98 (c.u.);
- increase of δ SDANN more than 2.05 (ms);
- increase in δ RMSSD more than 19.37 (ms);
- decrease of δ pNN50 less than 0.13 (%).

In a similar way, it is possible to characterize the changes of other indicators, which have probable differences in directionality and severity of changes during the performance of a breathing maneuver.

## 4 Discussion

A feature of this study is the use of discrete indicators of simultaneous measurements of the activity of the cardiovascular and respiratory systems. Of course, this limits the search for direct correlations of interaction that allow understanding physiological and pathophysiological aspects, but it allows, based on the search for the relations of discrete indicators, taking into account known and new functional tests, to identify diagnostically important features of cardiorespiratory relationships (Baumert et al., 2013; Baumert et al., 2015; Noskin et al., 2020; Matić et al., 2022; Parviainen et al., 2022).

The problem of diagnosing the state of athletes in the conditions of the educational and training process requires the use of express, valid, non-invasive methods of instrumental diagnostics of the functional state of the body, which would allow studying the impact of psychophysical loads directly during training and competitions (Thompson et al., 2015; Bringard et al., 2017; Flatt et al., 2017; Chen et al., 2021). For this purpose, heart rate monitors (Polar, Finland), MetaMax 3B devices (Cortex, Germany) are widely used for this purpose, the results of which are used to assess the current state and observe the power of loads during the educational and training process (according to indicators of changes in heart rate and HRV), monitoring of dynamics aerobic capacity (according to VO_2_ indicators) (Buchheit, 2014; Nakamura et al., 2017; Esco et al., 2018; Schneider et al., 2019; Hoffmann et al., 2020). The device we used (SACR) has the ability to simultaneously register and process indicators of the activity of the cardiorespiratory system. It is mobile relative to the place of research, so it was widely used by us in “field conditions”. On the other hand, the continued development of devices or body clothing would record indicators of the body’s activity to assess the functional state of the body not only in athletes (Mühlen et al., 2021), but also in ordinary people, as well as patients with various pathologies, especially cardiovascular and respiratory systems.

The main task of this study was to determine the changes in the indicators of the cardiorespiratory system when performing a simple breathing maneuver with a change in breathing rate for their possible further use in the practice of current control of athletes.

The analysis of pattern breathing data during the breathing maneuver showed that in comparison with spontaneous breathing, when performing controlled breathing with a frequency of 0.1 and 0.25 Hz, the indicators of V_T_/T_E_ (L×s^-1^) and V_T_/T_I_ (L×s^-1^) significantly increase. This increase led to a significant increase in V (L×min^-1^) at CR_6_ from 7.818 (6.307; 9.583) to 11.050 (7.930; 14.755), *p* = 0.000, and at CR_15_ to 13.740 (10.109; 19.447), *p* = 0.000. This made it possible to characterize breathing as liquid deep (CR_6_) and deepened (CR_15_). We considered the last type of breathing as a possible predictor of hyperventilation, because the calculated VO_2_ (L×min^-1^) increased almost 2 times from 0.360 (0.290; 0.441) to 0.632 (0.465; 0.895), *p* = 0.000 in comparison with SR. It should also be mentioned, which in previous studies by Bernardi et al. (2001) showed that 0.1 Hz RR suppressed both hypoxic and hypercapnic chemoreflex responses compared with spontaneous or controlled 0.25 Hz respiration.

We did not find data on the spectral analysis of breathing pattern parameters in the literature. Taking into account the capabilities of the SACR device and the automatic spectral analysis of inspiratory airflow rate indicators, we performed a spectral analysis of short recordings, which allowed us to calculate VRV indicators. Analyzing the results obtained during the performance of the breathing maneuver, it is possible to claim a decisive role in the modulation of the heart rate and blood pressure variability. Above in Figure 3 shows how HRV and BPV indicators change synchronously with breathing variability at SR, CR 0.1 Hz and CR 0.25 Hz, and in Figure 4 – how the absolute values of HR and SBP change in the respiratory cycle. The TP_R_ indicator, (L×min^-1^)^2^, which determines the total power of respiration, is closely related to the V (L×min^-1^) indicator, so its significant increase at CR 0.25 Hz is quite obvious, when V (L×min^-1^) is the largest. Currently, it is difficult to interpret the frequency characteristics of the spectrum, but they reflect the performance of the breathing maneuver. It should be mentioned that the use of these indicators clearly allowed to differentiate individuals with a high and low VO_2_max level (Guzii and Romanchuk, 2017) according to TP_R_, (L×min^-1^)^2^ and HF_R_, (L×min^-1^)^2^ indicators. The data of the examination of individuals with different types of heart rhythm regulation proved to be informative (Romanchuk and Guzii, 2020), the response to intense physical load in athletes with overstrain was differentiated according to the sympathetic and parasympathetic type (Romanchuk, 2022; Guzii et al., 2023). It was shown that an increase in LF_R_, (L×min^-1^)^2^ indicated parasympathetic overstrain (Romanchuk and Guzii, 2020). On the other hand, the decrease in the TP_R_ (L×min^-1^)^2^ indicator, after loads clearly characterized the athlete’s recovery after the competition (Romanchuk and Guzii, 2020). Differences in VRV indicators were also shown in controlled and uncontrolled bronchial asthma (Romanchuk et al., 2019) and in obese individuals (Bazhora and Romanchuk, 2018). The informativeness of this approach in the control of athletes with asthma due to physical load can be quite expected, which is important both in terms of diagnosis and in terms of preventing asthma attacks.

The data of the average values of the PQRST complex recorded a significant increase in HR (min^-1^) against the background of an increase in QTc (s). Changes in HR (min^-1^) at CR_6_ and CR_15_ are compensatory in response to an increase in preload of the heart due to an increase in blood return to the heart, while the dynamics of changes in QTc (s) may indicate certain overstrain of the contractile function. The dynamics of the latter can probably be informative with regard to the diagnosis of hidden signs of heart failure. However, this has yet to be proven.

Changes in HRV during the breathing maneuver demonstrated the well-known effect of increasing sinus arrhythmia during deep liquid breathing (CR_6_), which is completely eliminated at CR_15_. Taking into account the dynamics of other components of HRV, it can be stated that the effects of resonant increase of the LF component of HRV at CR 0.1 Hz do not fully correspond to resonant changes of the LF component of HRV at CR 0.25 Hz. The possible effect of hyperventilation suppresses vagal stimulation of the sinus node, which is reflected both by a significant increase in HR (min^-1^) and by an insufficient increase in the HF component of HRV. Separately, it should be noted a greater suprasegmental (VLF, ms^2^) effect on heart rate regulation at CR 0.1 Hz compared to SR, although they do not differ compared to CR 0.25 Hz. This allows us to assume almost the same involvement of subcortical structures during controlled breathing. The analysis of statistical and geometric indicators of HRV made it possible to establish that the ABI and RMSSD indicators, which are less sensitive to the activation of sympathetic influences at CR 0.25 Hz, and the pNN50 indicator, which is less sensitive to the suppression of sympathetic influences at CR 0.1 Hz, may have a certain differential value. From these positions, the results obtained during the formation of overstrain by sympathetic and parasympathetic types deserve attention (Guzii et al., 2020).

The measurement of absolute BP values by the SACR device has certain caveats regarding compliance with the values measured using the shoulder cuff, although in recent developments the measurement is being standardized. However, a significant decrease in SBPf (mmHg), DBPf (mmHg) and PBPf mmHg was found based on the results of BP measurement in the sitting position during the breathing maneuver. More attention was paid to the analysis of changes in SBP and DBP variability indicators. Regarding their analysis, without focusing on the absolute values, we will consider the variability of these indicators during the execution of the maneuver, because there were attempts to normalize them, but the non-normalized position regarding the measurement methodology lays down a certain discrepancy in the results related to SBP variability (Castiglioni et al., 2009). We did not find normalization results for DBP variability at all. We performed normalization, but only young people were concerned (Romanchuk, 2006; Guzii et al., 2019).

Without delving into the mechanisms, we can state that the changes of some indicators during the performance of the breathing maneuver are not differentiated. The response of VLF_SBP_, VLF_DBP_ and HF_DBP_ at CR 0.1 Hz and at CR 0.25 Hz was of the same type. At the same time, HF_SBP_ at CR 0.25 Hz was significantly greater than at SR and CR 0.1 Hz. The mechanism of its increase is the fastest resonant to the HF heart rate, however, we also indicated a certain inconsistency in comparison with the LF component from the side of this indicator. Perhaps this indicates the disappearance of Traube-Hering waves (Towie et al., 2012; Shantsila et al., 2015). On the other hand, changes in LF_SBP_ (mmHg^2^) and LF_DBP_ (mmHg^2^) are related to respiration and transmitted through HR (Tan and Taylor, 2010). In a previous study, it was shown that VLF_SBP_, VLF_DBP_, LF_SBP_, and LF_DBP_ indicators during SR increase under the influence of intense physical load during the formation of sympathetic overstrain and remain the same until the next day. At the same time, there is a decrease in HF_SBP_ immediately after exercise, but it is not persistent and is restored until the next morning. On the other hand, it was the increase in HF_SBP_ after exercise and its persistence until the next morning that indicated vagotonic overstrain, which was also accompanied by an increase in VLF_DBP_ immediately and its decrease below initial values the next morning (Guzii et al., 2021; Guzii et al., 2023).

During the breathing maneuver, fairly characteristic changes in BR_LF_ (ms×mmHg^-1^) and BR_HF_ (ms×mmHg^-1^) indicators occur. At CR 0.1 Hz, the indicators significantly increase, and at CR 0.25 Hz, there is a significant decrease below the SR level, which reflects the characteristics of baroreceptor mechanisms (Fadel, 2008; Fadel and Raven, 2012). It should be added that the results obtained by us in the study of highly qualified athletes with signs of sympathetic and parasympathetic overstrain development under the influence of intense physical load showed that BR_LF_ (ms×mmHg^-1^) and BR_HF_ (ms×mmHg^-1^) during SR in athletes with both types the overstrain decreases immediately after the load, and the next day it does not recover to the initial values (Guzii et al., 2021; Guzii et al., 2023). A study of athletes in a competitive process showed differences in recovery of BR_LF_ (ms×mmHg^-1^) and BR_HF_ (ms×mmHg^-1^) at CR 0.1 Hz and at CR 0.25 Hz (Guzii and Romanchuk, 2016). That is, it is quite logical to search for differences in the reaction of BR_LF_ (ms×mmHg^-1^) and BR_HF_ (ms×mmHg^-1^) in response to various stimuli, which can reveal additional mechanisms of baroreceptor sensitivity implementation in various human conditions.

There are many publications relating cardiorespiratory integration to measures of systemic hemodynamics (Eckberg and Karemaker, 2009; Elstad, 2012; Dempsey and Smith, 2019; Fisher et al., 2022), which use different parameterization approaches. Perhaps this characterizes certain differences in the results, especially related to the estimation of absolute parameters. The method we used was based on the calculation of the average discrete values of the measured intervals and segments of the PQRST complex. According to the data of our research, it was shown that performing a breathing maneuver with a change in the frequency of breathing has a significant effect on a number of indicators. At CR 0.1 Hz, in comparison with SR, there is no change in EDV (cm^3^), an increase in ESV (cm^3^), CO (dm^3^), CI (dm^3^×m^-2^) on the background of a decrease in SV (cm^3^), GPVR (dyn/s/cm^−5^) and SI (cm^3^×m^-2^). At CR 0.25 Hz, in comparison with SR, the unchanged ESV (cm^3^), an increase in CO (dm^3^), CI (dm^3^×m^-2^) against the background of a decrease in EDV (cm^3^), SV (cm^3^), GPVR (dyn/s/cm^−5^) and SI (cm^3^×m^-2^). That is, performing a breathing maneuver increases the preload on the heart, which is compensated by an increase in HR (min^-1^) against a background of a decrease in SBPf (mmHg). At the same time GPVR (dyn/s/cm^−5^) decreases. We recorded an increase in QTc (s), especially at a CR of 0.25 Hz in healthy young men, suggesting that performing such a maneuver may be useful in identifying hidden variants of heart contractile dysfunction, such as cardiomyopathies of various geneses.

Taking into account the ranges of increments of the specified HRV indicators during the breathing maneuver, the limits of the absolute values of the indicators, which can testify to increased and decreased reactivity of the body to the influence of controlled breathing compared to spontaneous breathing, are determined (Table 12). In this case, the possibility of taking into account minor deviations of reactivity within the limits of low expected values is demonstrated. Of course, this type of analysis requires refinement taking into account the initial values of the considered parameters, but it provides an opportunity to evaluate the functional state of the athlete’s body, at least, according to three characteristics. We are talking about the assessment of the initial state, taking into account the known normative values of the parameters at rest in the sitting position, and by the indicators of the increase in the indicators in response to controlled breathing during the breathing maneuver. Estimates of the values of these indicators, which will undergo clustering taking into account deviations from the expected values, can be used for the general characterization of the athlete’s condition.

In the end, it should be added that the main premise of this study was the lack of data in the literature on the combined registration of data on the activity of the cardiorespiratory system when performing such a breathing maneuver and the possibility of conducting such a study in field conditions. Therefore, further analysis of the differences in changes in indicators taking into account gender, age, direction and intensity of physical exertion in different periods of the training cycle can be, in our opinion, informative regarding the diagnosis, differentiation and forecasting of the development of states of functional and non-functional overreaching, as well as overtraining in the conditions of current control without significantly distracting the athlete from the training and competition processes.

## 5 Conclusion

According to the results of the study, it is shown that when performing a breathing maneuver with a change in the frequency of breathing, there are significant changes in cardiorespiratory parameters, the analysis of the increments of which made it possible to determine the directions of the dynamics of changes, their absolute values and informative limits regarding the possible occurrence of dysregulation of cardiorespiratory interactions.

## Data Availability

The original contributions presented in the study are included in the article/Supplementary Materials, further inquiries can be directed to the corresponding author.
