# Supplementary material for: Cardiorespiratory dynamics during respiratory maneuver in athletes
Source: Front Netw Physiol. 2023 Oct 30;3:1276899. doi: 10.3389/fnetp.2023.1276899 (PMC10643240; doi:10.3389/fnetp.2023.1276899)
Supplement: Supplementary file 1 [file Table1.DOCX]

Table 1

Distribution of athletes by sports and place of investigate

| Kind of sport | N | Examination place | |
| --- | --- | --- | --- |
| Long distance runners | 2 | “Olympyets”, Odesa School of Higth Sport Mastery | School doctor's office |
| Middle distance runners | 6 | “Olympyets”, Odesa School of Higth Sport Mastery | School doctor's office |
| Rowers on kayaks | 5 | FSC "Khimik", Pivdennyy water and rowing base | Base doctor's office |
| Rowers on canoes | 6 | FSC "Khimik", Pivdennyy water and rowing base | Base doctor's office |
| Table tennis players | 14 | TTC “Burevisnyk”, Odesa | TTC doctor's office |
| Boxing | 33 | Olympic educational sport center “Koncha Zaspa” | A small gym |
| Karate | 12 | SC “SUNPU”, Odesa | SC doctor's office |
| Freestyle wrestling | 11 | SC “SUNPU”, Odesa | SC doctor's office |
| Judo | 3 | SC “SUNPU”, Odesa | SC doctor's office |
| Greco-Roman wrestling | 5 | SC “SUNPU”, Odesa | SC doctor's office |
| Volleyball | 13 | SC “SUNPU”, Odesa | SC doctor's office |
| Water polo | 14 | WPC “Dinamo”, Lviv | WPC doctor's office |
| Handball | 9 | SC “SUNPU”, Odesa | SC doctor's office |
| Soccer | 35 | FC “Chernomorets”, Odesa | Base doctor's office |
| Gymnasts | 3 | SC “SUNPU”, Odesa | SC doctor's office |
| Acrobats | 2 | SC “SUNPU”, Odesa | SC doctor's office |
| Shooters | 10 | “Olympyets”, Odesa School of Higth Sport Mastery | School doctor's office |

Abbreviations: SC – sports club, FC – Soccer club, WPC – Water polo club, TTC – table tennis club, SUNPU – South Ukrainian National Pedagogical University

Table 2

Morphofunctional parameters of the examined group of athletes, Med (Q1;Q3), n= 183

| Indicator | Values |
| --- | --- |
| Body weigh, kg | 73.0 (68.0; 80.0) |
| Body length, cm | 178.0 (173.0; 182.0) |
| BMI, kg×m^-2^ | 23.5 (22.0; 24.8) |
| Body square, m^2^ | 1.90 (1.82; 2.01) |
| Body length (sitting), cm | 93.0 (91.0; 95.0) |
| Fat, % | 14.5 (11.3; 17.3) |
| Chest circumference (pause), cm | 97.0 (93.0; 102.0) |
| Chest excursion, cm | 8.0 (7.0; 9.0) |
| VC, ml | 4900.0 (4500.0; 5500.0) |
| δVC, % | 9.56 (-1.03; 21.04) |
| VI, ml×kg^-1^ | 66.7 (60.6; 72.9) |
| Test Shtange, s | 76.0 (63.0; 93.0) |
| Test Genchee, s | 40.0 (32.0; 50.0) |
| SBP, mmHg | 114 (110.0; 120.0) |
| DBP, mmHg | 70.0 (60.0; 80.0) |
| PBP, mmHg | 50.0 (40.0; 50.0) |

Abbreviations: BMI, body mass index; VC, vital lung capacity; δVC, different between vital lung capacity and proper vital lung capacity in percentage; VI, vital index; SBP, systolic blood pressure (by Korotkoff); DBP, diastolic blood presure (by Korotkoff); PBP, pulse blood pressure (by Korotkoff)

Table 3

Indicators of breathing pattern of the examined group of athletes during the breathing maneuver, Med (Q1;Q3), n= 183

| Indicator | SR | CR_6_ | CR_15_ | p | | |
| --- | --- | --- | --- | --- | --- | --- |
|  |  |  |  | SR-CR_6_ | SR-CR_15_ | CR_6_-CR_15_ |
| Ti, s | 1.76 (1.44; 2.09) | 4.98 (4.82; 5.12) | 1.92 (1.78; 2.06) | 0.000 | 0.004 | 0.000 |
| Te, s | 2.43 (2.04; 3.06) | 5.02 (4.90; 5.14) | 2.07 (1.95; 2.20) | 0.000 | 0.000 | 0.000 |
| V_T_, L | 0.570 (0.440; 0.700) | 1.700 (1.220; 2.270) | 0.890 (0.660; 1.280) | 0.000 | 0.000 | 0.000 |
| V_T_/T_E_, L×s^-1^ | 0.22 (0.17; 0.28) | 0.31 (0.24; 0.43) | 0.40 (0.29; 0.60) | 0.000 | 0.000 | 0.000 |
| V_T_/T_I_ , L×s^-1^ | 0.32 (0.27; 0.38) | 0.41 (0.29; 0.55) | 0.53 (0.38; 0.71) | 0.000 | 0.000 | 0.000 |
| T_I/_/T_TOT_ | 0.41 (0.39; 0.44) | 0.49 (0.47; 0.51) | 0.48 (0.46; 0.51) | 0.000 | 0.000 | 0.541 |
| RR, min^-1^ | 14.2 (12.0; 16.8) | 6.2 (6.0; 6.4) | 15.2 (15.0; 15.3) | 0.000 | 0.000 | 0.000 |
| V, L×min^-1^ | 7.818 (6.307; 9.583) | 11.050 (7.930; 14.755) | 13.740 (10.109; 19.447) | 0.000 | 0.000 | 0.000 |
| VO_2_,L×min^-1^ | 0.360 (0.290; 0.441) | 0.508 (0.365; 0.679) | 0.632 (0.465; 0.895) | 0.000 | 0.000 | 0.000 |

Abbreviations: Ti, mean inspiratory time; Te, mean expiratory time; V_T_, tidal volume; V_T_/T_E_, mean exspiratory flow; V_T_/T_I_, mean inspiratory flow; T_I_//T_TOT_, mean inspiratory duty cycle; RR, respiratory rate; V, minute ventilation; VO_2_, oxygen uptake.

Table 4

Indicators of volume respiration variability of the examined athletes during the breathing maneuver, Med (Q_1_; Q_3_), n= 183

| Indicator | SR | CR_6_ | CR_15_ | p | | |
| --- | --- | --- | --- | --- | --- | --- |
|  |  |  |  | SR-CR_6_ | SR-CR_15_ | CR_6_-CR_15_ |
| TP_R_, (L×min^-1^)^2^ | 349.7  (234.1; 547.6) | 655.4  (334.9; 1246.1) | 1108.9  (533.6; 2284.8) | 0.000 | 0.000 | 0.000 |
| VLF_R_, (L×min^-1^)^2^ | 2.9  (1.7; 4.0) | 10.2  (6.8; 18.5) | 5.3  (2.6; 10.2) | 0.000 | 0.000 | 0.000 |
| LF_R_, (L×min^-1^)^2^ | 16.0  (9.6; 56.3) | 542.9  (259.2; 1024.0) | 20.3  (10.9; 38.4) | 0.000 | 0.649 | 0.000 |
| LF_R_n,n.u. | 4.7  (2.5; 16.6) | 85.0  (78.0; 88.0) | 1.8  (1.3; 2.5) | 0.000 | 0.000 | 0.000 |
| HF_R_, (L×min^-1^)^2^ | 278.9  (169.0; 445.2) | 81.0  (47.7; 146.4) | 1062.8  (515.3; 2199.6) | 0.000 | 0.000 | 0.000 |
| HF_R_n,n.u. | 87.3  (74.2; 92.3) | 13.2  (10.2; 19.0 ) | 95.3  (92.9; 96.7) | 0.000 | 0.000 | 0.000 |
| LFHF_R_, (L×min^-1^)^2^/(L×min^-1^)^2^ | 0.053  (0.029; 0.203) | 6.452  (4.040; 8.585) | 0.020  (0.014; 0.029) | 0.000 | 0.000 | 0.000 |
| IC_R_, (L×min^-1^)^2^/(L×min^-1^)^2^ | 0.065  (0.035; 0.249) | 6.572  (4.113; 8.879) | 0.025  (0.018; 0.035) | 0.000 | 0.000 | 0.000 |

Abbreviations: TP_R_, dispersion of V_T_/T_E_ in a given time interval (total power) ≈≤ 0.4 Hz; VLF_R_, power spectrum of V_T_/T_E_ in the very-low frequency range ≤ 0.04 Hz; LF_R_, power spectrum of V_T_/T_E_ in the low frequency range 0.04-0.15 Hz; LF_R_n, LF_R_/(TP_R_ – VLF_R_)×100; spectrum power of V_T_/T_E_ in the low-frequency range in normalized units; HF_R_, power spectrum of V_T_/T_E_ in the high frequency range 0.15-0.4 Hz; HF_R_n, HF_R_/(TP_R_ – VLF_R_)×100; spectrum power of V_T_/T_E_ in the high-frequency range in normalized units; LFHF_R_, LF_R_[(L×min^-1^)^2^]/HF_R_[(L×min^-1^)^2^]; IC_R_, (LF_R_[(L×min^-1^)^2^]+VLF_R_[(L×min^-1^)^2^])/HF_R_[(L×min^-1^)^2^]; centralization index of respiration.

Table 5.

Informative changes in the indicators of the PQRST complex of the examined group of athletes during a breathing maneuver, Med (Q_1_; Q_3_), n= 183

| Indicator | SR | CR_6_ | CR_15_ | p | | |
| --- | --- | --- | --- | --- | --- | --- |
|  |  |  |  | SR-CR_6_ | SR-CR_15_ | CR_6_-CR_15_ |
| HR, min^-1^ | 67.2 (62.1; 76.9) | 71.0 (64.8; 78.8) | 76.9 (69.2; 89.3) | 0.000 | 0.000 | 0.000 |
| QTc, s | 0.408 (0.395; 0.422) | 0.412 (0.400; 0.424) | 0.421 (0.408; 0.434) | 0.000 | 0.000 | 0.000 |
| ST,n.u. | 0.089 (0.052; 0.136) | 0.102 (0.053; 0.148) | 0.103 (0.044; 0.152) | 0.005 | 0.289 | 0.557 |

Abbreviations: HR, heart rate; QTc, electrical systole corrected (by Bazett).

Table 6

Indicators of HRV of the examined athletes during the breathing maneuver, Med (Q1; Q3), n= 183

| Indicator | SR | CR_6_ | CR_15_ | p | | |
| --- | --- | --- | --- | --- | --- | --- |
|  |  |  |  | SR-CR_6_ | SR-CR_15_ | CR_6_-CR_15_ |
| TP, ms^2^ | 4096  (2450; 6939) | 18879  (13712; 25824) | 2884  (1747; 4900) | 0.000 | 0.000 | 0.000 |
| VLF, ms^2^ | 501.8  (249.6; 882.1) | 655.4  (408.0; 1049.8) | 547.6  (262.4; 998.6) | 0.001 | 0.083 | 0.142 |
| LF, ms^2^ | 1204.1  (645.2; 2642.0) | 15775.3  (11470.4; 20050.5) | 552.3  (306.3; 912.0) | 0.000 | 0.000 | 0.000 |
| LFn,n.u | 42.0  (24.8; 60.3) | 86.7  (81.9; 91.6) | 28.1  (17.7; 42.5) | 0.000 | 0.000 | 0.000 |
| HF, ms^2^ | 1664.6  (912.0; 3364.0) | 2043.0  (1069.3; 3636.1) | 1497.7  (645.2; 2745.8) | 0.405 | 0.001 | 0.000 |
| HFn,n.u. | 55.4  (35.8; 72.3) | 12.1  (7.6; 16.7) | 67.7  (53.3; 78.6) | 0.000 | 0.000 | 0.000 |
| LFHF, ms^2^/ms^2^ | 0.81  (0.36; 1.69) | 7.29  (4.84; 12.25) | 0.36  (0.25; 0.81) | 0.000 | 0.000 | 0.000 |
| IC_HR_, ms^2^/ms^2^ | 1.16  (0.53; 2.42) | 7.63  (5.12; 12.99) | 0.94  (0.51; 1.70) | 0.000 | 0.002 | 0.000 |

Abbreviations: TP, dispersion of RR intervals in a given time interval ≈≤ 0.4 Hz; (total power); VLF, power spectrum of HRV in the very-low frequency range ≤ 0.04 Hz; LF, power spectrum of HRV in the low frequency range 0.04-0.15 Hz; LFn, LF/(TP – VLF)×100; spectrum power of HRV in the low-frequency range in normalized units; HF, power spectrum of HRV in the high frequency range 0.15-0.4 Hz; HFn, HF/(TP – VLF)×100; spectrum power of HRV in the high-frequency range in normalized units; LFHF, LF[ms^2^]/HF[ms^2^]; IC_HR_, (LF[ms^2^]+ VLF[ms^2^])/HF[ms^2^]; centralization index of heart rate.

Table 7

Indicators of statistical and geometric analysis of HRV of the examined athletes during the breathing maneuver, Med (Q_1_; Q_3_), n= 183

| Indicator | SR | CR_6_ | CR_15_ | p | | |
| --- | --- | --- | --- | --- | --- | --- |
|  |  |  |  | SR-CR_6_ | SR-CR_15_ | CR_6_-CR_15_ |
| ABI, c.u. | 14.77 (9.65; 28.15) | 6.10 (4.01; 10.57) | 17.96 (9.83; 31.04) | 0.000 | 0.145 | 0.000 |
| SRAI, c.u. | 4.06 (2.90; 5.45) | 2.71 (2.12; 3.60) | 5.01 (3.55; 7.23) | 0.000 | 0.000 | 0.000 |
| ARI, c.u. | 4.75 (3.29; 7.68) | 3.57 (2.61; 5.29) | 5.87 (4.06; 8.91) | 0.000 | 0.007 | 0.000 |
| SI, c.u. | 84.10 (47.05; 162.27) | 39.24 (27.20; 64.41) | 119.59 (63.03; 206.78) | 0.000 | 0.001 | 0.000 |
| SDANN, ms | 61.82 (48.34; 83.49) | 130.81 (106.84; 152.11) | 51.95 (40.93; 69.00) | 0.000 | 0.000 | 0.000 |
| RMSSD, ms | 48.0 (33.5; 69.9) | 67.7 (48.3; 93.4) | 47.6 (33.2; 74.4) | 0.000 | 0.470 | 0.000 |
| pNN50, % | 12.5 (10.9; 14.9) | 12.5 (10.8; 22.7) | 11.8 (9.7; 18.0) | 0.813 | 0.000 | 0.000 |

Abbreviations: ABI, autonomic balance index; SRAI, subcortical regulation adequacy indicator; ARI, autonomic regulation index; SI, stress index; SDANN, standard deviation of the values of cardiointervals; RMSSD, square root of the sum of squares of the differences in the values of consecutive pairs of normal intervals; pNN50, the percentage of NN50 from the total number of consecutive pairs of intervals that differ by more than 50 milliseconds, obtained over the entire time recording.

Table 8

Changes in the average values of blood pressure from the finger cuff in the subjects of the examined athletes during the breathing maneuver, Med (Q_1_; Q_3_), n= 183

| Indicator | SR | CR_6_ | CR_15_ | p | | |
| --- | --- | --- | --- | --- | --- | --- |
|  |  |  |  | SR-CR_6_ | SR-CR_15_ | CR_6_-CR_15_ |
| SBPf, mmHg | 112.5 (110.0; 120.0) | 110.2 (104.0; 120.2) | 111.1 (102.3; 120.8) | 0.000 | 0.000 | 0.628 |
| DBPf, mmHg | 66.9 (52.4; 77.9) | 66.2 (45.4; 76.1) | 63.6 (48.1; 75.4) | 0.000 | 0.000 | 0.750 |
| PBPf, mmHg | 48.4 (41.2; 63.3) | 44.1 (37.0; 60.9) | 43.9 (36.8; 60.9) | 0.013 | 0.011 | 0.764 |

Abbreviations: SBPf, systolic blood pressure (from finger by Penaz); DBPf, diastolic blood presure (from finger by Penaz); PBPf, pulse blood pressure (from finger by Penaz).

Table 9

Changes in SBP and DBP variability indicators of the examined athletes during the breathing maneuver, Med (Q_1_; Q_3_), n= 183

| Indicator | SR | CR_6_ | CR_15_ | p | | |
| --- | --- | --- | --- | --- | --- | --- |
|  |  |  |  | SR-CR_6_ | SR-CR_15_ | CR_6_-CR_15_ |
| TP_SBP_, mmHg^2^ | 26.0 (16.0; 43.6) | 64.0 (39.7; 94.1) | 38.4 (23.0; 64.0) | 0.000 | 0.000 | 0.000 |
| VLF_SBP_, mmHg^2^ | 10.2 (4.4; 21.2) | 13.7 (6.3; 27.0) | 13.0 (6.3; 29.2) | 0.007 | 0.005 | 0.856 |
| LF_SBP_, mmHg^2^ | 6.8 (4.0; 10.9) | 36.0 (23.0; 57.8) | 6.3 (3.2; 11.6) | 0.000 | 0.629 | 0.000 |
| LF_SBP_n,n.u. | 52.9 (35.8; 68.8) | 88.9 (80.3; 93.6) | 31.1 (18.6; 45.2) | 0.000 | 0.000 | 0.000 |
| HF_SBP_, mmHg^2^ | 4.8 (2.9; 9.6) | 4.0 (2.3; 6.8) | 12.3 (7.3; 24.0) | 0.001 | 0.000 | 0.000 |
| HF_SBP_n,n.u. | 44.4 (29.2; 61.5) | 9.7 (5.9; 16.9) | 64.7 (52.1; 77.9) | 0.000 | 0.000 | 0.000 |
| LFHF_SBP_, mmHg^2^/mmHg^2^ | 1.19 (0.59; 2.34) | 9.18 (4.79; 15.92) | 0.48 (0.23; 0.88) | 0.000 | 0.000 | 0.000 |
| TP_DBP_, mmHg^2^ | 10.2 (6.8; 16.0) | 24.0 (15.2; 36.0) | 10.9 (6.8; 19.4) | 0.000 | 0.272 | 0.000 |
| VLF_DBP_, mmHg^2^ | 3.6 (2.0; 6.3) | 4.8 (2.6; 9.0) | 4.4 (2.3; 7.3) | 0.001 | 0.008 | 0.585 |
| LF_DBP_, mmHg^2^ | 4.0 (2.3; 6.8) | 14.4 (7.8; 23.0) | 2.6 (1.7; 4.4) | 0.000 | 0.000 | 0.000 |
| LF_DBP_n,n.u. | 74.4 (59.3; 81.7) | 85.6 (78.3; 91.2) | 57.3 (37.7; 74.9) | 0.000 | 0.000 | 0.000 |
| HF_DBP_, mmHg^2^ | 1.2 (0.6; 2.0) | 2.0 (1.2; 3.2) | 1.7 (1.0; 3.6) | 0.000 | 0.000 | 0.978 |
| HF_DBP_n,n.u. | 22.5 (15.5; 35.3) | 12.5 (8.0; 19.3) | 37.1 (22.8; 56.8) | 0.000 | 0.000 | 0.000 |
| LFHF_DBP_, mmHg^2^/mmHg^2^ | 3.35 (1.66; 5.34) | 6.81 (4.08; 11.29) | 1.54 (0.69; 3.28) | 0.000 | 0.000 | 0.000 |
| IC_SBP_, mmHg^2^/mmHg^2^ | 3.71 (1.36; 8.21) | 13.30 (7.84;25.04) | 1.57 (0.80; 3.09) | 0.000 | 0.000 | 0.000 |
| IC_DBP_, mmHg^2^/mmHg^2^ | 7.36 (4.20; 12.72) | 10.40 (5.81;17.84) | 4.30 (2.01; 9.18) | 0.000 | 0.000 | 0.000 |
| BR_LF_, ms×mmHg^-1^ | 14.2 (9.7; 20.1) | 20.4 (14.6; 26.7) | 9.4 (6.8; 12.9) | 0.000 | 0.000 | 0.000 |
| BR_HF_, ms×mmHg^-1^ | 18.3 (11.9; 27.5) | 20.8 (15.4; 30.4) | 10.3 (6.2; 15.6) | 0.000 | 0.000 | 0.000 |

Abbreviations: TP_SBP_, dispersion of SBPf in a given time interval (total power) ≈≤ 0.4 Hz; VLF_SBP_, power spectrum of SBPf in the very-low frequency range ≤ 0.04 Hz; LF_SBP_, power spectrum of SBPf in the low frequency range 0.04-0.15 Hz; LF_SBP_n, LF_SBP_/(TP_SBP_ – VLF_SBP_)×100; spectrum power of SBPf in the low-frequency range in normalized units; HF_SBP_, power spectrum of SBPf in the high frequency range 0.15-0.4 Hz; HF_SBP_n, HF_SBP_/(TP_SBP_ – VLF_SBP_)×100; spectrum power of SBPf in the high-frequency range in normalized units; LFHF_SBP_, LF_SBP_[mmHg^2^]/HF_SBP_[mmHg^2^]; TP_DBP_, dispersion of DBPf in a given time interval (total power) ≈≤ 0.4 Hz; VLF_DBP_, power spectrum of DBPf in the very-low frequency range ≤ 0.04 Hz; LF_DBP_, power spectrum of DBPf in the low frequency range 0.04-0.15 Hz; LF_DBP_n, LF_DBP_/(TP_DBP_ – VLF_DBP_)×100; spectrum power of DBPf in the low-frequency range in normalized units; HF_DBP_, power spectrum of DBPf in the high frequency range 0.15-0.4 Hz; HF_DBP_n, HF_DBP_/(TP_DBP_ – VLF_DBP_)×100; spectrum power of DBPf in the high-frequency range in normalized units; LFHF_SBP_, LF_SBP_[mmHg^2^]/HF_SBP_[mmHg^2^]; IC_SBP_, (LF_SBP_[mmHg^2^]+ VLF_SBP_[mmHg^2^])/HF_SBP_ [mmHg^2^]; centralization index of systolic blood pressure; IC_DBP_, (LF_DBP_[mmHg^2^]+ VLF_DBP_[mmHg^2^])/HF_DBP_[mmHg^2^]; centralization index of diastolic blood pressure; BR_LF,_ sensitivity of arterial baroreflex in low frequency range (√LF [ms^2^]×LF[mmHg^2^]^-1^); BR_HF_, sensitivity of arterial baroreflex in high frequency range (√HF [ms^2^]×HF[mmHg^2^]^-1^).

Table 10

Changes in the hemodynamic parameters of the examined athletes during the breathing maneuver, Med (Q_1_; Q_3_), n= 183

| Indicator | SR | CR_6_ | CR_15_ | p | | |
| --- | --- | --- | --- | --- | --- | --- |
|  |  |  |  | SR-CR_6_ | SR-CR_15_ | CR_6_-CR_15_ |
| EDV, cm^3^ | 95.9 (85.2; 109.2) | 96.6 (85.2; 109.0) | 94.4 (84.9; 105.3) | 0.741 | 0.000 | 0.000 |
| ESV, cm^3^ | 29.7 (23.5; 37.3) | 30.6 (24.4; 36.1) | 30.6 (24.1; 35.6) | 0.022 | 0.156 | 0.577 |
| SV, cm^3^ | 66.0 (60.3; 73.9) | 65.7 (60.1; 72.9) | 63.6 (57.9; 71.2) | 0.010 | 0.000 | 0.000 |
| CO, dm^3^ | 4.5 (4.1; 5.0) | 4.8 (4.3; 5.2) | 5.0 (4.5; 5.5) | 0.000 | 0.000 | 0.000 |
| CI, dm^3^×m^-2^ | 2.39 (2.16; 2.62) | 2.50 (2.26; 2.78) | 2.59 (2.33; 2.94) | 0.000 | 0.000 | 0.000 |
| GPVR, dyn/s/cm^−5^ | 1577 (1409; 1740) | 1486 (1363; 1628) | 1417 (1288; 1585) | 0.000 | 0.000 | 0.000 |
| SI, cm^3^×m^-2^ | 34.9 (31.1; 38.8) | 34.3 (31.2; 38.2) | 33.2 (29.9; 37.2) | 0.017 | 0.000 | 0.000 |

Abbreviations: EDV, end-diastolic volume; ESV, end-systolic volume; SV, stroke volume; CO, cardiac output; CI, cardiac index; GPVR, general periferical vascular resistance; SI, stroke index.

Table 11

Changes in indicators of synchronization of cardiorespiratory interaction in the examined athletes during the breathing maneuver, Med (Q_1_; Q_3_), n= 183

| Indicator | SR | CR_6_ | CR_15_ | p | | |
| --- | --- | --- | --- | --- | --- | --- |
|  |  |  |  | SR-CR_6_ | SR-CR_15_ | CR_6_-CR_15_ |
| VSI, dm^3^× L^-1^ | 0.586 (0.477; 0.745) | 0.441 (0.327; 0.582) | 0.360 (0.247; 0.477) | 0.000 | 0.000 | 0.000 |
| Hildebrandt index | 4.77 (3.98; 6.20) | 10.92 (9.97; 12.14) | 5.00 (4.48; 5.84) | 0.000 | 0.209 | 0.000 |

Abbreviations: VSI, CO (dm^3^)/V (L×min^-1^); volume synchronization index; Hildebrandt index, HR (min^-1^)/RR (min^-1^), rate synchronization index.

Table 12

Increments of indicators in the examined athletes during the breathing maneuver at CR_6_ and CR_15_ compared to SR, Med (Q_1_; Q_3_), n= 183

|  | δ SR – CR_6_ | δ SR – CR_15_ | z | p |
| --- | --- | --- | --- | --- |
| δ HR, min^-1^ | 2.3 (0.2; 5.9) | 8.1 (4.3; 13.4) | 11.3 | 0.000 |
| δ QTc, s | 0.004 (-0.002; 0.010) | 0.013 (0.005; 0.019) | 9.9 | 0.000 |
| δ ST,n.u. | 0.008 (-0.014; 0.030) | 0.005 (-0.027; 0.032) | 0.5 | 0.652 |
| δ TP,ms^2^ | 13918.5 (7681.4; 19471.6) | -1003.2 (-3419.4; 327.4) | 11.7 | 0.000 |
| δ VLF,ms^2^ | 146.7 (-230.7; 585.6) | 51.1 (-330.3; 456.0) | 1.5 | 0.142 |
| δ LF,ms^2^ | 13683.4 (8402.0; 18107.7) | -656.5 (-1762.6; -108.0) | 11.7 | 0.000 |
| δ LFn,n.u | 44.3 (23.6; 60.6) | -8.7 (-26.8; 1.4) | 11.7 | 0.000 |
| δ HF,ms^2^ | 26.7 (-1019.2; 1437.2) | -216.6 (-1156.3; 428.0) | 4.4 | 0.000 |
| δ HFn,n.u. | -42.7 (-59.5; -22.4) | 8.6 (-2.6; 26.0) | 11.7 | 0.000 |
| δ LFHF,ms^2^/ms^2^ | 5.89 (3.60; 10.12) | -0.20 (-1.19; 0.00) | 11.7 | 0.000 |
| δ TP_SBP_, mmHg^2^ | 29.7 (9.0; 58.6) | 9.8 (-6.2; 31.2) | 6.4 | 0.000 |
| δ TP_DBP_, mmHg^2^ | 13.0 (4.6; 23.4) | 0.9 (-5.0; 5.9) | 9.7 | 0.000 |
| δ VLF_SBP_, mmHg^2^ | 3.0 (-6.5; 13.4) | 2.8 (-5.8; 15.1) | 0.2 | 0.855 |
| δ VLF_DBP_, mmHg^2^ | 1.0 (-1.4; 3.7) | 1.0 (-1.8; 4.0) | 0.5 | 0.584 |
| δ LF_SBP_, mmHg^2^ | 28.4 (13.6; 49.0) | -0.3 (-4.4; 4.0) | 11.2 | 0.000 |
| δ LF_DBP_, mmHg^2^ | 9.7 (4.6; 16.8) | -0.6 (-2.9; 0.6) | 11.5 | 0.000 |
| δ LF_SBP_n, n.u. | 34.1 (13.8; 50.4) | -18.3 (-34.1; -2.2) | 11.7 | 0.000 |
| δ LF_DBP_n, n.u. | 11.1 (3.0; 23.4) | -13.3 (-28.1; -1.5) | 11.5 | 0.000 |
| δ HF_SBP_, mmHg^2^ | -0.8 (-4.6; 1.3) | 6.2 (1.4; 16.1) | 10.6 | 0.000 |
| δ HF_DBP_, mmHg^2^ | 0.7 ( 0.0; 1.9) | 0.4 ( -0.3; 2.0) | 0.03 | 0.976 |
| δ HF_SBP_n, n.u. | -32.2 (-49.0; -11.5) | 18.5 (2.4; 32.7) | 11.7 | 0.000 |
| δ HF_DBP_n, n.u. | -8.3 (-20.9; -2.4) | 11.6 (0.8; 26.0) | 11.4 | 0.000 |
| δ LFHF_SBP_, mmHg^2^/mmHg^2^ | 6.72 (2.77; 13.65) | -0.49 (-1.50; -0.07) | 11.7 | 0.000 |
| δ LFHF_DBP_, mmHg^2^/mmHg^2^ | 3.24 (0.86; 7.07) | -1.28 (-2.82; -0.09) | 11.3 | 0.000 |
| δ TP_R_, (L×min^-1^)^2^ | 256.1 (45.3; 711.5) | 710.4 (194.4; 1684.6) | 9.9 | 0.000 |
| δ VLF_R_, (L×min^-1^)^2^ | 7.0 (3.2; 15.4) | 2.2 (0.0; 6.6) | 7.8 | 0.000 |
| δ LF_R_, (L×min^-1^)^2^ | 478.7 (215.8; 972.2) | 2.2 (-24.5; 18.2) | 11.7 | 0.000 |
| δ LF_R_, n, n.u. | 75.0 (59.1; 82.9) | -2.5 (-14.1; -0.7) | 11.7 | 0.000 |
| δ HF_R_, (L×min^-1^)^2^ | -185.1 (-326.3; -74.9) | 722.4 (219.5; 1744.9) | 11.7 | 0.000 |
| δ HF_R_, n, n.u. | -70.5 (-79.7; -53.0) | 7.4 (2.1; 20.0) | 11.7 | 0.000 |
| δ LFHF_R_,(L×min^-1^)^2^/(L×min^-1^)^2^ | 6.11 (3.48; 8.29) | -0.03 (-0.18; -0.01) | 11.7 | 0.000 |
| δ T_I_,s | 2.5 (1.7; 2.9) | -0.0 (-0.4; 0.3) | 11.7 | 0.000 |
| δ T_E_,s | 2.9 (2.3; 3.6) | -0.3 (-0.9; 0.1) | 11.7 | 0.000 |
| δ V_T_, L | 1.10 (0.70; 1.69) | 0.32 (0.12; 0.68) | 11.1 | 0.000 |
| δ V_T_/T_I_, L×s^-1^ | 0.09 (0.02; 0.17) | 0.20 (0.10; 0.36) | 8.6 | 0.000 |
| δ V_T_/T_E_, L×s^-1^ | 0.08 (-0.01; 0.22) | 0.21 (0.08; 0.37) | 10.1 | 0.000 |
| δ Ti/(Ti+Te), c.u. | 0.03 (-0.02; 0.07) | 0.03 (-0.01; 0.07) | 2.5 | 0.011 |
| δ RR, min^-1^ | -7.7 (-10.3; -5.5) | 1.1 (-1.5; 3.4) | 11.7 | 0.000 |
| δ V, L×min^-1^ | 3.0 (0.9; 6.2) | 6.1 (3.0; 10.4) | 9.2 | 0.000 |
| δ VO_2_, | 0.14 (0.04; 0.28) | 0.28 (0.14; 0.48) | 9.2 | 0.000 |
| δ EDV, cm^3^ | 0.0 (-3.8; 3.5) | -1.8 (-6.4; 1.8) | 4.3 | 0.000 |
| δ ESV, cm^3^ | 0.4 (-1.2; 2.4) | 0.4 (-1.7; 2.3) | 0.6 | 0.577 |
| δ SV, cm^3^ | -0.6 (-2.8; 1.7) | -2.2 (-5.3; 0.1) | 6.7 | 0.000 |
| δ CO, dm^3^ | 0.2 (0.0; 0.4) | 0.4 (0.1; 0.7) | 7.0 | 0.000 |
| δ CI, dm^3^×m^-2^ | 0.103 (0.000; 0.203) | 0.206 (0.059; 0.353) | 7.0 | 0.000 |
| δ GPVR, dyn/s/cm^−5^ | -78.6 (-170.4; 6.5) | -131.0 (-262.3; -32.2) | 6.0 | 0.000 |
| δ SI, cm^3^×m^-2^ | -0.22 (-1.49; 0.89) | -1.12 (-2.85; 0.10) | 6.4 | 0.000 |
| δ VSI, dm× L ^-1^ | -0.128 (-0.255; -0.051) | -0.229 (-0.358; -0.128) | 8.1 | 0.000 |
| δ Hildebrandt index | 6.02 (4.98; 7.26) | 0.28 (-0.82; 1.08) | 11.7 | 0.000 |
| δ BR_LF_, ms×mmHg^-1^ | 5.05 (0.57; 10.15) | -4.32 (-9.11; -1.52) | 11.6 | 0.000 |
| δ BR_HF_, ms×mmHg^-1^ | 3.31 (-3.89; 9.20) | -6.91 (-11.86; -2.82) | 10.5 | 0.000 |
| δ IC_HRV_, ms^2^/ms^2^ | 5.98 (3.37; 10.92) | -0.13 (-1.11; 0.41) | 11.7 | 0.000 |
| δ IC_SBP_, mmHg^2^/mmHg^2^ | 8.18 (2.69; 19.93) | -1.52 (-6.36; 0.10) | 11.2 | 0.000 |
| δ IC_DBP_, mmHg^2^/mmHg^2^ | 2.04 (-2.23; 7.74) | -2.44 (-6.65; 1.03) | 8.0 | 0.000 |
| δ IC_R_,(L×min^-1^)^2^/(L×min^-1^)^2^ | 6.20 (3.58; 8.46) | -0.04 (-0.22; -0.01) | 11.7 | 0.000 |
| δ ABI, c.u. | -7.74 (-18.16; -1.70) | 0.74 (-6.85; 11.94) | 9.9 | 0.000 |
| δ SRAI, c.u. | -1.16 (-2.08; -0.39) | 1.06 (0.03; 2.11) | 11.4 | 0.000 |
| δ ARI, c.u. | -1.17 (-3.45; 0.26) | 0.51 (-1.18; 2.37) | 8.0 | 0.000 |
| δ SI, c.u. | -37.67 (-105.23; -7.31) | 14.52 (-26.51; 90.98) | 10.4 | 0.000 |
| δ SDANN, ms | 59.53 (41.02; 79.39) | -10.47 (-24.62; 2.05) | 11.7 | 0.000 |
| δ RMSSD, ms | 13.63 (0.26; 35.57) | -0.43 (-13.94; 19.37) | 5.9 | 0.000 |
| δ pNN50, % | -0.26 (-1.29; 7.80) | -0.94 (-2.35; -0.13) | 5.5 | 0.000 |

Abbreviations: see above.
