# Supplementary figures and images for: Cardiorespiratory dynamics during respiratory maneuver in athletes
Source: Front Netw Physiol. 2023 Oct 30;3:1276899. doi: 10.3389/fnetp.2023.1276899 (PMC10643240; doi:10.3389/fnetp.2023.1276899)

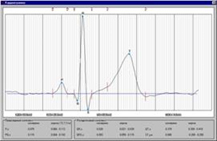

Supplement: Supplementary file 2 [file Image1.JPEG]
